# Prospective Evaluation of First-Line Erlotinib in Advanced Non-Small Cell Lung Cancer (NSCLC) Carrying an Activating EGFR Mutation: A Multicenter Academic Phase II Study in Caucasian Patients (FIELT)

**DOI:** 10.1371/journal.pone.0147599

**Published:** 2016-03-31

**Authors:** Jacques De Grève, Jan Van Meerbeeck, Johan F. Vansteenkiste, Lore Decoster, Anne-Pascale Meert, Peter Vuylsteke, Christian Focan, Jean-Luc Canon, Yves Humblet, Guy Berchem, Benoit Colinet, Danny Galdermans, Lionel Bosquée, Joanna Vermeij, Alex Dewaele, Caroline Geers, Denis Schallier, Erik Teugels

**Affiliations:** 1 Department of Medical Oncology, Oncologisch Centrum, Universitair Ziekenhuis Brussel, Brussels, Belgium; 2 Department of Thoracic Oncology, University Hospital, Gent, Belgium; 3 Department of Pneumology, University Hospital KU Leuven, Leuven, Belgium; 4 Department of Pneumology, Institut Jules Bordet, Brussels, Belgium; 5 Department of Medical Oncology, Clinique et Maternité Sainte-Elisabeth, Namur, Belgium; 6 Department of Medical Oncology, CHC Clinique Saint Joseph, Liège, Belgium; 7 Department of Medical Oncology, Grand Hôpital de Charleroi, Charleroi, Belgium; 8 Department of Medical Oncology, Centre du Cancer de l'Université Catholique de Louvain, Brussels, Belgium; 9 Department of Medical Oncology, Centre Hospitalier du Luxembourg, Luxembourg, Luxembourg; 10 Department of Medical Oncology, ZNA Middelheim Hospital, Antwerp, Belgium; 11 Department of Respiratory Medicine, CHU Sart-Tilman, Liège, Belgium; 12 Department of Medical Oncology, ZNA Jan Palfijn, Merksem, Belgium; Catalan Institute of Oncology, SPAIN

## Abstract

**Introduction:**

Epidermal Growth Factor Receptor (EGFR) tyrosine kinase inhibition is the preferred first-line treatment of advanced adenocarcinoma of the lung that harbors EGFR activating tyrosine kinase domain mutations. Most data available pertain to Asian populations in which such mutations are more prevalent. We report on the long-term results of first-line treatment with erlotinib in Caucasian patients with advanced adenocarcinoma of the lung that have a somatic EGFR mutation in their tumor.

**Methods:**

Multicenter academic prospective phase II study with erlotinib in patients with an activating EGFR tyrosine kinase (TK) domain somatic mutation (any exon encoding the kinase domain) in the tumor and no prior treatment for their advanced disease.

**Results:**

Phenotypic preselecting of 229 patients led to a high EGFR mutation detection rate of 24% of which 46 patients were included in the phase II study. With a progression free survival (PFS) of 81% at three months the study met its primary endpoint for presumed superiority over chemotherapy. With an overall median PFS of 11 months and a median overall survival (OS) of 23 months, the results compare favorably with results obtained in randomized studies using TKI in first line in EGFR mutation positive adenocarcinoma of the lung.

**Conclusion:**

The present study reinforces the use of EGFR tyrosine kinase inhibition (TKI) as a first line treatment of choice for advanced adenocarcinoma of the lung carrying an activating EGFR mutation. The mutation rate in preselected Caucasian patients is higher than previously reported. Issues relevant for clinical practice are discussed.

**Trial Registration:**

ClinicalTrials.gov NCT00339586

## Introduction

Patients with advanced non-small cell lung cancer (NSCLC) are incurable with a low probability for long-term survival. With platinum-based doublet chemotherapy a response rate of around 25% and a median OS of about 10–12 months can be obtained in metastatic disease [1] corresponding to a PFS of 60% or less at 3 months [2]

A novel approach to the treatment of advanced NSCLC was introduced with the use of agents blocking the tyrosine kinase part of the Epidermal Growth Factor Receptor (EGFR). Some patients had dramatic responses to these EGFR tyrosine kinase inhibitors (TKI’s) [3, 4]. Ten years ago it became clear that mutations in the exons coding for the intracellular EGFR kinase domain, in particular in exon 19 and 21 highly increase the sensitivity to EGFR TKI’s [5, 6]. These mutations have been observed in 10% or less of all lung cancers tested, in 30% of adenocarcinoma of the lung if the smoking history was maximally 15 years and up to 50% in never-smokers [7], although these figures depend highly on the ethnicity of the population tested, being much higher in East-Asian populations than in Caucasians.

Most (90%) sensitizing mutations are found in exon 19 and 21. Mutations in exon 20 are generally not associated with increased sensitivity towards reversible TKI’s [8]. The overall response rate (ORR) to TKI in EGFR mutant lung cancers varies between 60 and 90% [9].

Gefitinib in an Asian population [10, 11], and erlotinib, in both a Caucasian [12] and an Asian [13] population, were validated as superior to chemotherapy in terms of PFS in patients whose tumors harbor sensitizing driver mutations in the EGFR gene and are therefore recommended as the preferred first-line therapies for these patients.

FIELT (First line Inhibitor of EGFR in Lung cancer Treatment) is a prospective academic study investigating the efficacy and tolerability of first-line treatment with erlotinib in newly diagnosed advanced adenocarcinoma of the lung carrying EGFR kinase domain mutations, as well as the feasibility of inserting genomic testing in a multicenter clinical setting (S1 Text). The study aimed to estimate whether first-line erlotinib could reach an efficacy threshold higher than chemotherapy.

At the time of initiation of FIELT in 2006, advanced lung cancer was treated indiscriminately with platinum-based chemotherapy and no data were available on the prospective first-line use of any EGFR TKI in phenotypically or genotypically selected NSCLC, while only retrospective data were available for gefitinib [14].

## Materials and Methods

The study was an academic study registered at clinicaltrials.gov as NCT00339586 (S1 Text).

### Patient eligibility

Key eligibility criteria were locally advanced or metastatic (Stage IIIB or Stage IV) adenocarcinoma of the lung. Radiotherapy and adjuvant or neo-adjuvant chemotherapy completed more than six months before inclusion were allowed. Patients should not have received previous chemotherapy for metastatic disease and had to have a smoking history of less than 15 years and have stopped smoking more than one year before diagnosis.

Measurable disease was not mandatory. An ECOG performance status of 0–3 was required. Previously diagnosed and treated central nervous system metastases or spinal cord compression with evidence of stable disease for at least two months was permitted. Specific exclusion criteria were: pre-existing symptomatic interstitial lung disease, not related to the current malignancy, and gastrointestinal disease or concomitant food or drug intake which could impair absorption and metabolism of erlotinib. Significant ophthalmological abnormalities, especially severe dry eye syndrome, keratoconjunctivitis sicca, Sjögren syndrome, severe exposure keratitis or any other disorder likely to increase the risk of corneal epithelial lesions were also exclusion criteria.

Standard phase II selection criteria were applicable for organ function. Separate signed informed consents were required for mutation testing and subsequent inclusion in the erlotinib treatment phase.

### Study design and treatment

The study was a multicenter academic single arm phase II study in 17 university and non-university centers in Belgium and Luxemburg (NCT00339586).

The study was approved by the institutional medical ethics review board of each participating center. The study was approved by the ethics committee of the Academisch Ziekenhuis-Vrije Universiteit Brussel which was the leading ethics committee that according to Belgian law approved the study in a single opinion form. Academisch Ziekenhuis-Vrije Universiteit Brussel is the former name of the current UZ Brussel of the Vrije Universiteit Brussel (S2 Text). Participants provided a written informed consent.

### Mutation analysis

Patients had a central tumor EGFR mutation testing in the Laboratory of Medical and Molecular Oncology (LMMO) of the Oncologisch Centrum, UZ Brussel. Mutation analysis was performed on DNA extracted from three consecutive 10μm thick sections of formalin fixed and paraffin embedded material. Tissue sections were verified for the presence of a sufficient proportion of malignant cells and manually macro-dissected when necessary. The collected DNA was used to perform a hemi-nested PCR followed by a denaturing gradient gel electrophoresis (DGGE). The PCR/DGGE method requires only very small amounts of template DNA and is able to detect any mutation found in exon 18–21, but could not reliably detect mutations in samples in which the tumor DNA represented less than 25% of the total DNA. Mutations were confirmed by Sanger sequencing, but to minimize waiting time for physicians, patients could be entered in the treatment phase of the trial, based on the DGGE results. No results had to be revoked based on the subsequent Sanger sequence. Results were delivered to the participating clinicians within two weeks except for cases in which the material was insufficient to obtain a reliable result in a first round. If a second analysis was needed for such inconclusive results, the responsible physician was informed of the delay.

### Treatment phase

Upon ascertainment of an EGFR mutation in the tumor sample, consenting patients started erlotinib treatment 150 mg daily plus best supportive care within one week. Any mutation found in exon 18–21 made the patient eligible for inclusion in the treatment phase of the study. Treatment was until disease progression (RECIST definition, both for patients with or without measurable lesions) or prohibitive toxicity. Response assessments (CT imaging and physical) and ancillary parameters were scheduled at 6, 12, 18 and 24 weeks after treatment initiation, thereafter every 12 weeks.

Toxicity was scored every four weeks during treatment according to the common toxicity criteria adverse events version 3.0 [15].

The primary endpoint of the study was to establish a significant clinical benefit by achieving at least a 70% PFS rate at three months on erlotinib. Secondary objectives and endpoints were ORR according to RECIST criteria, response duration under erlotinib treatment, the effect on Quality of Life (QOL), ECOG performance score, weight, the PFS and OS. The QOL results will be reported elsewhere. The analysis of the primary efficacy endpoint was based on all subjects who received at least one dose of study treatment. Data analysis cut-off was done in May 2013. If scheduled follow-up evaluations were missing, patients were considered progressive, unless a later examination confirmed persistent remission.

The premises for the sample size determination were as follows. With platinum based chemotherapy, a PFS of 60% or less is obtained at 3 months follow-up in advanced NSCLC [1, 16]. Taking into account the known general better tolerability of erlotinib it was proposed that if a PFS of more than 70% could be obtained at three months, then this treatment deserved further evaluation in the first-line treatment of advanced NSCLC. If the study would result in a PFS less than 50% at three months, single agent erlotinib should not be further evaluated in this setting as outcomes inferior in terms of efficacy to the current standard were not acceptable.

### Statistical considerations

Based on these premises, a one-step Fleming design required at least 33 patients to be included in the study (α = 10%; β = 10%). With the provision of a margin for eligibility/evaluation issues, a prospective number to be included was set at 40 patients.

PFS and OS were estimated from the date of registration until respectively the documentation of progression and the date of death, irrespective of the cause of death. Patients who had not progressed or died at the time of the analysis were censored at the date of last contact. PFS and OS were calculated according to the Kaplan-Meier method with SPSS statistical software (version 20.0; SPSS Inc., Chicago, IL). Results are presented with 95% confidence intervals (CIs).

## Results

### Mutation analysis

From January 2006 through March 2010, tumor tissue from 229 phenotypically selected patients was analyzed for the presence of EGFR kinase domain mutations. Tissue area ranged from 1 mm^2^ to 600 mm^2^, with 40% of the sections smaller than 5 mm^2^ (mostly needle biopsies).

**Fig 1:**
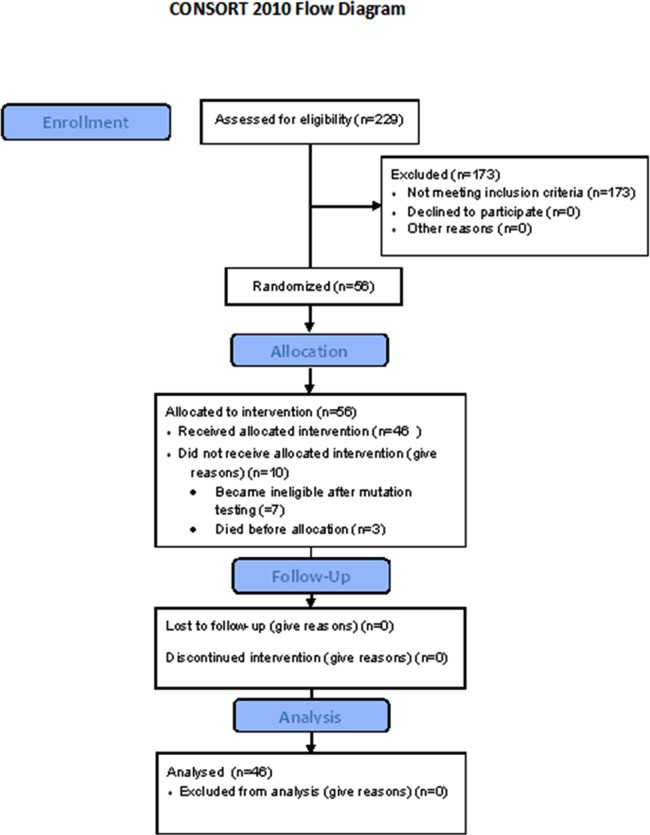
FIELT study flow.

Baseline material was insufficient for analysis in 24/229 samples (10%) due to the lack of adequate material (almost no tissue material available, no tumor cells in tissue, no amplifiable DNA). An EGFR kinase domain mutation was found in 56 out of 205 evaluable patients (27% or 24% of the original phenotypically selected population) (Table 1). Five mutations were previously unreported (two in exon 19, three in exon 20). The samples in which the tumor DNA was less than 25% of the total DNA represented 30% of all samples. Although we stated to the referring clinical investigators that a negative result should be considered as unreliable in these cases, we did find mutations in 23% of these cases, a similar proportion as in the overall population. A subsequent analysis with a more sensitive PNA (Peptide Nucleic Acid) PCR method [17] showed four more mutations (7% of all mutations). The overall sensitivity of the mutation detection method used in this study was thus 93% in evaluable samples or 90% in all samples, also considering the pathology limitations (absence of analyzable material). Other mutations found were: KRAS in 16%, BRAF in 2%, HER2 in 2%, HER3 in 0.4%, all mutually exclusive. The FIELT study flow is detailed in Fig 1.

**Table 1:** Mutations found.

Exon	Mutations		Times found (in COSMIC)	DXS/COBAS*
	Nucleotide/s Change	Amino acid/s Change		
**18**	c.2156G>C	p.Gly719Ala	1 (31x)	yes/yes
**19**	c.2235_2249del	p.Glu746_Ala750del	22 (785x)	yes/yes
	c.2236_2250del	p.Glu746_Ala750del	3 (360x)	yes/yes
	c.2237_2257delinsTCT	p.Glu746_Pro753delinsValSer	1 (2x)	no/yes
	c.2237_2253delinsTTCCT	p.Glu746_Thr751delinsValPr	1 (2x)	no/no
	c.2239_2248delinsC	p.Leu747_A750del insPro	1 (77x)	yes/yes
	c.2240_2254del	p.Leu747_Thr751del	1 (51x)	yes/yes
	c.2240_2257del	p.Leu747_Pro753delins Ser	2 (121x)	yes/yes
	c.2248_2276delinsCCAAC	p.Ala750_Ile759delinsProThr	1 (0x)	no/no
	c.2249_2277delinsGAAGT	p.Ala750_Ile759del insGlySer	1 (0x)	no/no
	c.2253_2276del	p.Ser752_Ile759del	1 (5x)	no/no
**20**	c.2303_2311dup9	p.Ser768_Asp770dup	1 (1x)?	no/no
	c.2319_2320insAACCAC	p.Pro772_His773insHisAsn	1 (0x)	no/no
	c.2311delAinsGTCC	p.Asn771del insValHis	1 (0x)	no/no
	c.2311_2312insCCA	p.Asp770_Asn771insThr	1 (0x)	no/no
	c.2310_2311insGGT	p.Asp770_Asn771insGly	1 (x5)	yes/yes
**21**	c.2573T>G	p.Leu858Arg	15 (1607x)	yes/yes

*indicates whether mutation is listed as detectable by Dxs Therascreen or COBAS.? = mutation that supposedly has been misnamed in the COSMIC database

Ten patients (18%) in whom an EGFR mutation was found did not enter the phase II part of the study because of various reasons: three became ineligible between an inconclusive first mutation screen and identification of the EGFR mutation in a second sample, three patients died before inclusion, one deteriorated and became ineligible and in three patients the reasons are unknown.

### Patient characteristics

The characteristics of the 46 patients included in the phase II study are provided in Table 2. Eighty five percent of patients were female. The median age was 72 yrs. (35–83 yrs.) and 37% of the patients were aged 75 or older. Two patients were included although not strictly fulfilling the selection criteria: one patient had a 25 pack-year of cigarette smoking history, but had since stopped smoking for 30 years and received therefore a waiver and one patient was stage IB (T2N0M0) recurrent disease after prior curative intent radiotherapy and was not any longer eligible for local curative intent treatment. Thirty eight were never smokers, seven were past smokers and one was a current smoker.

**Table 2:** Characteristics of the 46 patients included in the phase II study and treated with erlotinib.

**Age**	72 median (35–83 yrs.)
**Performance status**	PSO = 11
	PS1 = 28
	PS2 = 6
	PS3 = 1
**Stage**	Stage IB = 1
	Stage IIIB = 4
	Stage IV = 41
**Sex**	7 male;39 female
**EGFR Mutation**	Exon 19 = 27
	Exon 21 = 15
	Exon 20 = 3
	Exon 18 = 1
**Cigarette smoking history (pack years)**	Median 0 yrs. (0–25 yrs.)

### Efficacy

The efficacy results are reported on intent to treat basis including the two ineligible patients. A separate analysis with these two patients removed did not alter the results.

**Fig 2:**
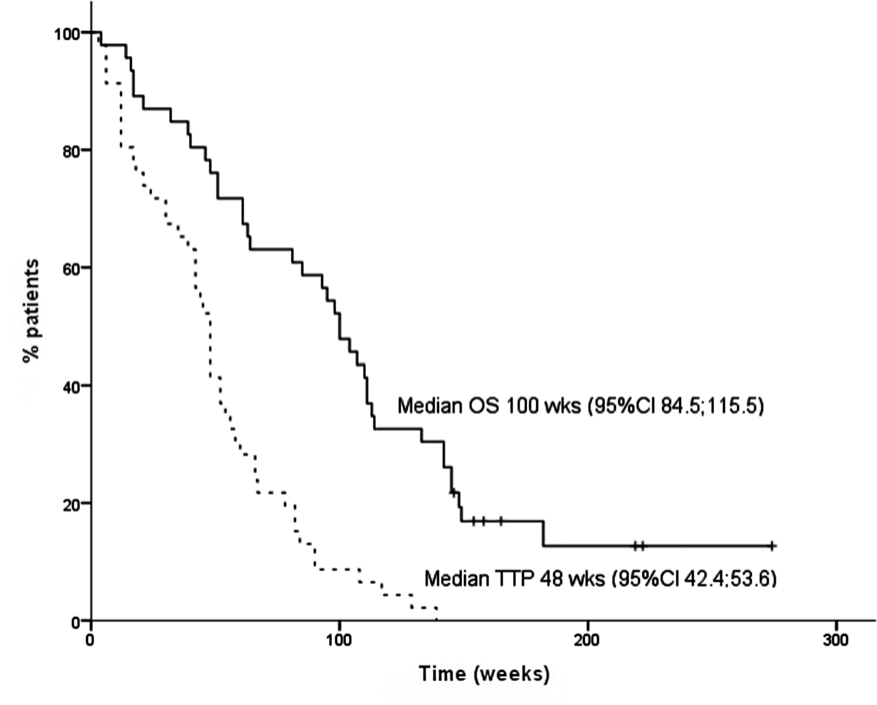
Survival outcome. Kaplan-Meier curves for overall and progression free survival of the FIELT cohort.

The PFS rate (PFSR) at three months was 81% and at six months 72%. The median PFS was 11 months (95% CI = 9.7–12.3 months) (Fig 2).

Twenty six patients achieved a partial remission (PR) (57%), one a complete remission (CR) (2%); ten a stable disease (SD) (22%) and nine had progressive disease (PD) as best response (20%). Although this was not the primary endpoint, responses were confirmed by a second measurement at least four weeks later. The clinical benefit rate (CR+PR+SD) was thus 81%. The distribution of responses was similar in exon 19 and 21 mutations.

Median duration of response was 9.7 months (2.7–29 + months) (+ means patient alive beyond); 9.7 months for cases with objective remissions (2.7–29+ months) and 10.3 months for those with stable disease (4.1–23.5 months).

With a median follow-up from treatment initiation of 45 months (36–84 months), median OS was 23 months (95% CI = 21.3–28.6+ months) (Fig 2). For patients achieving an objective response (PR or CR), median OS was 23.9 months (3.9–52.5 months), for patients that with SD as best response 14.5 months (3.2–39.6 months) and for progressing patients 3.9 months (0.9–9.2 months).

There was a numeric difference in OS between PS 0/1 and 2/3 patients: median 23 months (95%CI = 20–26 months) vs 13 months (95%CI = 7–21 months). The difference is statistically not significant according to log rank test p = 0.687, probably due to the small numbers.

Median overall survival was 25 months (95%CI = 20–29 months) for the younger patients and 15 months (95%CI = 0–30 months) OS for the elderly patients with a p-value of 0.222. Despite the numeric difference there is extensive overlap in outcomes.

A similar non-significant ratio was observed with a cut-off of 70 years (used in geriatrics) with a median OS of 26 months (95%CI = 23–28 months) vs 20 months (95%CI = 10–29 months)

At the time of analysis six patients were alive with median treatment duration on erlotinib of 18 months (11–32 months) of which three had been switched to gefitinib for tolerance reasons.

Median treatment duration was 12.8 months (0.6–33 months) and average treatment duration was 13.3 months. When we also consider the patients that were eligible, but never received erlotinib the median treatment duration is 10.4 months (0–33 months).

Eight patients (17%) (Table 3) failed within 12 weeks on erlotinib, (median PFS 2.8 months) of treatment initiation, and they were therefore considered as being de novo resistant to erlotinib. These patients had a much shorter OS of 5.6 months (1–26 months) compared to the overall 24 months median OS of the other patients (p< 0.002, two-sided). One patient however had a survival of 26 months despite an erlotinib treatment that lasted only four months, reflecting the impact of subsequent chemotherapy in this patient population. Factors that could explain a lack of response are an exon 20 mutation for two patients [18] and a heavy smoking history for one patient. Other past smokers benefited from erlotinib treatment albeit less than the overall study population: one 15 pack year: one 8 month PFS; one 5 pack year: one 18 month PFS; one 1 pack year, immediate progression.

**Table 3:** Characteristics of patients that failed early on erlotinib.

Initials	Sex	Age	EGFR mutation	Smoking status	Survival (months)
180 CV	F	73	EX 19 (p.Glu746_Ala750del)	Never	0.9
173 LAL	F	72	EX 20 (p.Asn771del insValHis)*	Never	3.2
229 FM	F	64	EX 19 (p.Glu746_Ala750del)	Never	3.7
241 DEL	M	78	EX 21 p.Leu858Arg	25 pack year	3.9
224 FPM	F	42	EX 19 p.Leu747_Pro753delins Ser	Never	7.4
208 VEC	F	48	EX 18 p.Gly719Ala	One year	9.2
42 TF	F	38	EX 20 p.Ser768_Asp770dup**	Never	14
8 MI	F	35	EX 21 p.Leu858Arg	Never	26.2

* Novel mutation

** reported once in COSMIC database; all other mutations are recurrent.

PY: pack years; F: female; M: male; ex: exon

Some never smokers with an exon 19 deletion mutation were also de novo resistant (Table 3).

One patient with an exon 18 mutation that is generally sensitive to EGFR TKI [19], failed immediately, received one second line of therapy and survived for 9 months.

### Post-study treatment

All patients had stopped erlotinib treatment at the time of analysis. Second- and further- lines of treatment were as reported by the participating centers. Twenty-nine (64%) of the 45 patients for whom the information was available received one or more second or further line treatments. For sixteen patients (36%) no second-line treatment was reported. For one patient the information was not available. Seventeen of the 29 (59%; 38% of total) received only one second-line therapy, nine (31%) received also a third-line and three (10%) also received also a fourth-line. Of the 29 patients who did receive further therapy, 17 received a targeted agent (7 gefitinib, 9 afatinib, one continued erlotinib beyond progression).

Seventeen patients received chemotherapy, of which ten a platinum-based doublet. Seven patients received only (sequential) single agent chemotherapies. Three of these patients were more than 75 years old. Four patients received radiotherapy to the brain (three) and the chest (one).

### Safety

Treatment with erlotinib was generally well tolerated with manageable toxicity as reported by the participating investigators (Table 4). One patient died without disease progression and one stopped because of skin toxicity while still responding after 18.9 months of treatment and subsequently received gefitinib with a continued sustained response and good tolerance.

**Table 4:** Common toxicities.

	Grade 1	Grade 2	Grade 3
Skin rash	14	17	10
Diarrhea	16	14	4
Ocular toxicity	10	11	0

## Discussion

At the time of initiation of the current phase II study no prospective data were available about the efficacy of EGFR TKI in EGFR mutant lung cancers. This has changed dramatically since, as several randomized studies have shown that both gefitinib and erlotinib as reversible EGFR inhibitors and afatinib as a covalent pan-HER inhibitor outperform chemotherapy as a first-line therapy in these patients with regard to PFS and QOL [10–13]. Most of the studies have been performed in Asian or predominantly Asian populations. Only a few studies have been conducted in homogeneous Caucasian populations, and the current study is the only one to supplement the data generated with erlotinib in the EURTAC study [12].

The two most representative landmark studies are the iPASS study with gefitinib in an Asian population [11] and the EURTAC study with erlotinib in a European Caucasian population [12]. More recently, similar results were obtained in the LUX-Lung 3 study with the irreversible pan-HER inhibitor afatinib [20]. Afatinib resulted in a major improvement in PFS versus the optimal standard chemotherapy (pemetrexed and cisplatin) for this type of lung cancer. No survival benefit has generally been observed in these studies, attributed to the important crossover to TKI as a second-line treatment in the chemotherapy arms, except for afatinib in patients with a del19 EGFR mutations in the tumor [21].

The PFSR at three months of 81% in the current study exceeds the primary endpoint of the study that hypothesized a PFSR of > 70%.

The inclusion of 10% of mutations in exon 20 that are less sensitive to EGFR TKI [22] makes our population less favorable than in the two randomized studies with erlotinib that included only patients with sensitizing mutations. Despite this, the PFS of 11 months in our study is intermediate between the PFS of 13 months in the Optimal study [23] and the PFS of 9.7 months in the EURTAC study [12]. In the more recent Lux-Lung 3 study with afatinib that also included other mutations than exon 19/21, the PFS was 11.1 months, similar to our outcome [20].

The median OS was 23 months in the current study, 22 months in the Optimal and 19.3 months in the EURTAC study. The current phase II study thus further clarifies the exact impact of erlotinib in Caucasian patients with an EGFR mutation-positive adenocarcinoma, indicating that erlotinib has a similar therapeutic impact in Caucasian as in Asian patients.

Treatment beyond progression was limited as median treatment duration was approximately two months longer than the PFS, similar to the OPTIMAL study. This occurred despite the protocol specification that patients could be treated until progression and indicates clinical benefit beyond progression.

Of interest, older patients benefited as much as younger patients. Most of these patients might otherwise not have benefited from optimal doublet chemotherapy and consequently the availability of EGFR targeted treatments has a large impact on this population introducing an effective treatment for a previously unmet medical need. This is in line with other data obtained with gefitinib [24].

The mutation detection rate was high (24%) in this phenotypically preselected population. In a more diluted general Caucasian lung cancer population the mutation rate is 10% or less. In the EURTAC study in a population similar to ours, the mutation rate (only exons 19 and 21) was 16.6%. Today, in routine practice upfront reflex EGFR mutation testing should be performed in all patients with adenocarcinoma of the lung and in squamous cell lung cancer with a non-smoking history.

We used a generic method for mutation testing that covered all exons encoding the kinase domain of EGFR. This method had a high sensitivity of 93% similar to the sensitivity of commercial kits such as Cobas® and Dxs Therascreen®. In the current cohort five novel mutations were identified, not present in the Cosmic data-base (http://cancer.sanger.ac.uk/cancergenome/projects/cosmic/). Several unique mutations would possibly not have been detected by the DxS Therascreen (9/56) and Cobas (8/56) commercial diagnostic mutation detection kits (Table 1). This includes sensitizing mutations which would have been denied an EGFR TKI therapy when using these commercial kits.

The small proportion of false negatives (7%) is not overlapping between both approaches: in the commercial kits some not listed sensitizing mutations can be missed but these methods can detect highly diluted mutations whereas our method was limited with regard to the dilution of the mutant DNA, which we proved subsequently by using a more sensitive technique. For the same reason, our method was unable to identify minority mutations such as the T790M, which at that time seemed of minor importance for the first-line therapy as it had no impact on the therapeutic strategy. This has changed with the documentation of a negative prognostic impact [25] and with the development of novel third generation EGFR inhibitors that can inhibit the T790M EGFR and their exploration in first-line setting [26].

The few false negatives with either method are unacceptable when one considers the therapeutic implications. Therefore, we and others have switched to an amplicon-based deep sequencing method (Roche 454 technology, Roche Diagnostics Belgium) that allows maximal detection of mutations and also allows simultaneous examination of other drugable genes without having to resort to sequential diagnostics. It is thereby important that all EGFR kinase domain exons are included in the analysis.

In the 10% of patients in whom biopsies are quantitatively or qualitatively insufficient, repeat biopsies in metastatic sites (immediately or later in the disease course) should be done as soon as feasible and acceptable to the patient. It is important that this specific recommendation is included in the report to the clinician whenever this might be relevant. An alternative would be to develop the collection and molecular analysis of circulating tumor cells which, for lung cancer, is not yet optimally developed. Analyzing plasma DNA (cfDNA) has been validated as a more efficient and straightforward method for the diagnosis of EGFR mutations in patients in whom a tissue diagnosis is not possible [27].

The incomplete translation of mutation detection to treatment in a prospective setting is surprising as 18% eligible patients did not enter the treatment phase with erlotinib. A similar drop-out rate (16%) has also been observed in the phase II study by Rosell et al [28]. In the EURTAC study 10% of patients did not receive erlotinib per protocol after randomization [12]. In the Chinese Optimal study, 99% of those allocated to erlotinib received at least one dose of study drug [13], a remarkable compliance that might have a cultural basis.

Seventeen percent of the patients failed early and were considered as being de novo resistant to erlotinib. The median OS for these patients was much shorter, but not necessarily in each patient. Indeed one patient, treated in second- line with carboplatin-gemcitabine survived for 26 months, illustrating the complementarity of therapeutics for these patients. There are several mechanisms of de novo resistance to EGFR TKI [29], but an explanation cannot be found in all patients.

We were unable to identify characteristics that could predict TKI resistance in most of these patients. Three patients with an exon 20 mutation did not do well, with two failing early and one achieving SD but with an OS of only 9 months. But there were also three non-smokers with an exon 19 mutation that failed early. In one series a KRAS mutation was found to coexist with EGFR mutation in 2/40 cases [29], but this was not the case in our patients. One of these patients was a heavy and current smoker who failed immediately. We hypothesize that in smokers the EGFR mutation occurs in a context in which a large number of cigarette induced mutations also drive the malignant phenotype and dilute the pathogenic and therapeutic relevance of the mutant EGFR. There is also evidence that in current smokers nicotine diminishes the effectiveness of EGFR-TKI [30].

Further genomic studies could in the future bring to light mechanisms of de novo and acquired resistance and prompt the investigation of upfront combined therapeutic strategies.

The proportion of patients receiving a second-line treatment was low (only 33% chemotherapy and 28% TKI). Only twelve patients received two or more lines of further treatment, one of which received five lines of further therapy and is still alive at 38+ months. For eighteen patients (39%) the local investigators reported no second-line systemic treatment. A similar observation was made in the Optimal study in which 31% of the patients in the erlotinib arm did not receive a second-line treatment [13].

These data are surprising in view of the relatively high efficacy of chemotherapy in such patients. It suggests that in practice erlotinib might be continued beyond progression until the disease rapidly deteriorates, the patient’s condition might become less eligible or the patient is less willing to be further treated with chemotherapy. The general consensus is that EGFR-TKI should be given upfront considering their tolerability compared to chemotherapy leading to a more prolonged preservation of QOL [31]. If the opportunity is missed in the first-line, then these patients should receive a TKI in any subsequent line, as early as possible. In patients in whom baseline genotyping was non-conclusive, attempts should be made to repeat biopsies and genotyping upon progression.

## Conclusion

In this prospective phase II study in phenotypically selected patients with a somatic EGFR mutation in their tumor erlotinib was well tolerated and highly effective in the majority of the patients. The study further clarifies the impact of first-line erlotinib in EGFR mutant lung cancer in Caucasian patients. Elderly patients seem to benefit from EGFR-TKI as well as younger patients, thus filling an important medical need.

In order to minimize the false negativity rate, the most sensitive method for mutation detection should be used (deep sequencing of all kinase domain exons) in order not to deny patients a TKI treatment. Mechanisms underlining primary resistance to EGFR TKI need further exploration.

## Supporting Information

S1 Text FIELT protocol version 2.0. (DOC)

S2 Text Ethical committee 05–122 Advice. (PDF)

S3 Text TREND Statement checklist. (PDF)

## References

[1] Bunn PA Jr. Treatment of advanced non-small-cell lung cancer with two-drug combinations. Journal of clinical oncology: official journal of the American Society of Clinical Oncology. 2002;20(17):3565–7. Epub 2002/08/31. . 12202651 10.1200/JCO.2002.20.17.3565

[2] Scagliotti GV, Parikh P, von Pawel J, Biesma B, Vansteenkiste J, Manegold C, et al Phase III study comparing cisplatin plus gemcitabine with cisplatin plus pemetrexed in chemotherapy-naive patients with advanced-stage non-small-cell lung cancer. Journal of clinical oncology: official journal of the American Society of Clinical Oncology. 2008;26(21):3543–51. Epub 2008/05/29. 10.1200/JCO.2007.15.0375 . 18506025

[3] Shepherd FA, Rodrigues Pereira J, Ciuleanu T, Tan EH, Hirsh V, Thongprasert S, et al Erlotinib in previously treated non-small-cell lung cancer. The New England journal of medicine. 2005;353(2):123–32. Epub 2005/07/15. 10.1056/NEJMoa050753 . 16014882

[4] Thatcher N, Chang A, Parikh P, Rodrigues Pereira J, Ciuleanu T, von Pawel J, et al Gefitinib plus best supportive care in previously treated patients with refractory advanced non-small-cell lung cancer: results from a randomised, placebo-controlled, multicentre study (Iressa Survival Evaluation in Lung Cancer). Lancet. 2005;366(9496):1527–37. Epub 2005/11/01. 10.1016/S0140-6736(05)67625-8 . 16257339

[5] Paez JG, Janne PA, Lee JC, Tracy S, Greulich H, Gabriel S, et al EGFR mutations in lung cancer: correlation with clinical response to gefitinib therapy. Science. 2004;304(5676):1497–500. Epub 2004/05/01. 10.1126/science.1099314 . 15118125

[6] Lynch TJ, Bell DW, Sordella R, Gurubhagavatula S, Okimoto RA, Brannigan BW, et al Activating mutations in the epidermal growth factor receptor underlying responsiveness of non-small-cell lung cancer to gefitinib. The New England journal of medicine. 2004;350(21):2129–39. Epub 2004/05/01. 10.1056/NEJMoa040938 . 15118073

[7] Pham D, Kris MG, Riely GJ, Sarkaria IS, McDonough T, Chuai S, et al Use of cigarette-smoking history to estimate the likelihood of mutations in epidermal growth factor receptor gene exons 19 and 21 in lung adenocarcinomas. Journal of clinical oncology: official journal of the American Society of Clinical Oncology. 2006;24(11):1700–4. Epub 2006/03/01. 10.1200/JCO.2005.04.3224 . 16505411

[8] Yasuda H, Park E, Yun CH, Sng NJ, Lucena-Araujo AR, Yeo WL, et al Structural, biochemical, and clinical characterization of epidermal growth factor receptor (EGFR) exon 20 insertion mutations in lung cancer. Science translational medicine. 2013;5(216):216ra177 Epub 2013/12/20. 10.1126/scitranslmed.3007205 24353160 PMC3954775

[9] Morita S, Okamoto I, Kobayashi K, Yamazaki K, Asahina H, Inoue A, et al Combined survival analysis of prospective clinical trials of gefitinib for non-small cell lung cancer with EGFR mutations. Clinical cancer research: an official journal of the American Association for Cancer Research. 2009;15(13):4493–8. Epub 2009/06/18. 10.1158/1078-0432.CCR-09-0391 . 19531624

[10] Mok TS, Wu YL, Thongprasert S, Yang CH, Chu DT, Saijo N, et al Gefitinib or carboplatin-paclitaxel in pulmonary adenocarcinoma. The New England journal of medicine. 2009;361(10):947–57. Epub 2009/08/21. 10.1056/NEJMoa0810699 . 19692680

[11] Fukuoka M, Wu YL, Thongprasert S, Sunpaweravong P, Leong SS, Sriuranpong V, et al Biomarker analyses and final overall survival results from a phase III, randomized, open-label, first-line study of gefitinib versus carboplatin/paclitaxel in clinically selected patients with advanced non-small-cell lung cancer in Asia (IPASS). Journal of clinical oncology: official journal of the American Society of Clinical Oncology. 2011;29(21):2866–74. Epub 2011/06/15. 10.1200/JCO.2010.33.4235 . 21670455

[12] Rosell R, Carcereny E, Gervais R, Vergnenegre A, Massuti B, Felip E, et al Erlotinib versus standard chemotherapy as first-line treatment for European patients with advanced EGFR mutation-positive non-small-cell lung cancer (EURTAC): a multicentre, open-label, randomised phase 3 trial. The lancet oncology. 2012;13(3):239–46. Epub 2012/01/31. 10.1016/S1470-2045(11)70393-X . 22285168

[13] Zhou C, Wu YL, Chen G, Feng J, Liu XQ, Wang C, et al Erlotinib versus chemotherapy as first-line treatment for patients with advanced EGFR mutation-positive non-small-cell lung cancer (OPTIMAL, CTONG-0802): a multicentre, open-label, randomised, phase 3 study. The lancet oncology. 2011;12(8):735–42. Epub 2011/07/26. 10.1016/S1470-2045(11)70184-X . 21783417

[14] Janne PA, Johnson BE. Effect of epidermal growth factor receptor tyrosine kinase domain mutations on the outcome of patients with non-small cell lung cancer treated with epidermal growth factor receptor tyrosine kinase inhibitors. Clinical cancer research: an official journal of the American Association for Cancer Research. 2006;12(14 Pt 2):4416s–20s. Epub 2006/07/22. 10.1158/1078-0432.CCR-06-0555 . 16857820

[15] Trotti A, Colevas AD, Setser A, Rusch V, Jaques D, Budach V, et al CTCAE v3.0: development of a comprehensive grading system for the adverse effects of cancer treatment. Seminars in radiation oncology. 2003;13(3):176–81. Epub 2003/08/07. 10.1016/S1053-4296(03)00031-6 . 12903007

[16] Smit EF, van Meerbeeck JP, Lianes P, Debruyne C, Legrand C, Schramel F, et al Three-arm randomized study of two cisplatin-based regimens and paclitaxel plus gemcitabine in advanced non-small-cell lung cancer: a phase III trial of the European Organization for Research and Treatment of Cancer Lung Cancer Group—EORTC 08975. Journal of clinical oncology: official journal of the American Society of Clinical Oncology. 2003;21(21):3909–17. Epub 2003/10/29. 10.1200/JCO.2003.03.195 . 14581415

[17] Shahi RB, De Brakeleer S, De Greve J, Geers C, In't Veld P, Teugels E. Detection of EGFR-TK Domain-activating Mutations in NSCLC With Generic PCR-based Methods. Diagnostic molecular pathology: the American journal of surgical pathology, part B. 2014 Epub 2014/02/04. 10.1097/PDM.0000000000000035 . 25751592

[18] Yasuda H, Kobayashi S, Costa DB. EGFR exon 20 insertion mutations in non-small-cell lung cancer: preclinical data and clinical implications. The lancet oncology. 2012;13(1):e23–31. Epub 2011/07/19. 10.1016/S1470-2045(11)70129-2 . 21764376

[19] Sharma SV, Bell DW, Settleman J, Haber DA. Epidermal growth factor receptor mutations in lung cancer. Nature reviews Cancer. 2007;7(3):169–81. Epub 2007/02/24. 10.1038/nrc2088 . 17318210

[20] Sequist LV, Yang JC, Yamamoto N, O'Byrne K, Hirsh V, Mok T, et al Phase III study of afatinib or cisplatin plus pemetrexed in patients with metastatic lung adenocarcinoma with EGFR mutations. Journal of clinical oncology: official journal of the American Society of Clinical Oncology. 2013;31(27):3327–34. Epub 2013/07/03. 10.1200/JCO.2012.44.2806 . 23816960

[21] Yang JC, Wu YL, Schuler M, Sebastian M, Popat S, Yamamoto N, et al Afatinib versus cisplatin-based chemotherapy for EGFR mutation-positive lung adenocarcinoma (LUX-Lung 3 and LUX-Lung 6): analysis of overall survival data from two randomised, phase 3 trials. The lancet oncology. 2015;16(2):141–51. Epub 2015/01/16. 10.1016/S1470-2045(14)71173-8 . 25589191

[22] Wu JY, Wu SG, Yang CH, Gow CH, Chang YL, Yu CJ, et al Cancer with epidermal growth factor receptor exon 20 mutations is associated with poor gefitinib treatment response. Clin Cancer Res. 2008;14(15):4877–82. 10.1158/1078-0432.CCR-07-5123 . 18676761

[23] Zhou C, Wu YL, Chen G, Feng J, Liu XQ, Wang C, et al Final overall survival results from a randomised, phase III study of erlotinib versus chemotherapy as first-line treatment of EGFR mutation-positive advanced non-small-cell lung cancer (OPTIMAL, CTONG-0802). Annals of oncology: official journal of the European Society for Medical Oncology / ESMO. 2015 Epub 2015/07/05. 10.1093/annonc/mdv276 . 26141208

[24] Tateishi K, Ichiyama T, Hirai K, Agatsuma T, Koyama S, Hachiya T, et al Clinical outcomes in elderly patients administered gefitinib as first-line treatment in epidermal growth factor receptor-mutated non-small-cell lung cancer: retrospective analysis in a Nagano Lung Cancer Research Group study. Medical oncology. 2013;30(1):450 Epub 2013/01/15. 10.1007/s12032-012-0450-2 . 23315220

[25] Ding D, Yu Y, Li Z, Niu X, Lu S. The predictive role of pretreatment epidermal growth factor receptor T790M mutation on the progression-free survival of tyrosine-kinase inhibitor-treated non-small cell lung cancer patients: a meta-analysis. OncoTargets and therapy. 2014;7:387–93. Epub 2014/03/14. 10.2147/OTT.S58870 24623981 PMC3949752

[26] Cortot AB, Janne PA. Molecular mechanisms of resistance in epidermal growth factor receptor-mutant lung adenocarcinomas. European respiratory review: an official journal of the European Respiratory Society. 2014;23(133):356–66. Epub 2014/09/02. 10.1183/09059180.00004614 . 25176972 PMC9487318

[27] Karachaliou N, Mayo-de Las Casas C, Queralt C, de Aguirre I, Melloni B, Cardenal F, et al Association of EGFR L858R Mutation in Circulating Free DNA With Survival in the EURTAC Trial. JAMA oncology. 2015;1(2):149–57. Epub 2015/07/17. 10.1001/jamaoncol.2014.257 . 26181014

[28] Rosell R, Moran T, Queralt C, Porta R, Cardenal F, Camps C, et al Screening for epidermal growth factor receptor mutations in lung cancer. The New England journal of medicine. 2009;361(10):958–67. Epub 2009/08/21. 10.1056/NEJMoa0904554 . 19692684

[29] Takeda M, Okamoto I, Fujita Y, Arao T, Ito H, Fukuoka M, et al De novo resistance to epidermal growth factor receptor-tyrosine kinase inhibitors in EGFR mutation-positive patients with non-small cell lung cancer. Journal of thoracic oncology: official publication of the International Association for the Study of Lung Cancer. 2010;5(3):399–400. Epub 2010/02/27. 10.1097/JTO.0b013e3181cee47e . 20186026

[30] Filosto S, Becker CR, Goldkorn T. Cigarette smoke induces aberrant EGF receptor activation that mediates lung cancer development and resistance to tyrosine kinase inhibitors. Molecular cancer therapeutics. 2012;11(4):795–804. Epub 2012/02/04. 10.1158/1535-7163.MCT-11-0698 . 22302097 PMC4370319

[31] Oizumi S, Kobayashi K, Inoue A, Maemondo M, Sugawara S, Yoshizawa H, et al Quality of life with gefitinib in patients with EGFR-mutated non-small cell lung cancer: quality of life analysis of North East Japan Study Group 002 Trial. The oncologist. 2012;17(6):863–70. Epub 2012/05/15. 10.1634/theoncologist.2011-0426 22581822 PMC3380886

