# Supplementary material for: Prospective Evaluation of First-Line Erlotinib in Advanced Non-Small Cell Lung Cancer (NSCLC) Carrying an Activating EGFR Mutation: A Multicenter Academic Phase II Study in Caucasian Patients (FIELT)
Source: PLoS One. 2016 Mar 31;11(3):e0147599. doi: 10.1371/journal.pone.0147599 (PMC4816447; doi:10.1371/journal.pone.0147599)
Supplement: S1 Text — (DOC) [file pone.0147599.s001.doc]

| **FIELT STUDY** |
| --- |
|  |

**F**irst line **I**nhibitor of **E**GFR in **L**ung cancer **T**reatment

**MULTICENTER STUDY**

**PROSPECTIVE EVALUATION OF SMALL MOLECULE EGFR-1 TYROSINE KINASE INHIBITION AS A FIRST-LINE TREATMENT IN PATIENTS WITH ADVANCED NON-SMALL CELL LUNG CANCER (NSCLC) HARBOURING A MUTANT EGFR GENE**

Amended version 2.0

16 September 2005

**Study Protocol**

**VUB O5-OO1**

**Study sponsor & coordination**

Dienst Medische Oncologie - Oncologisch Centrum

Academisch Ziekenhuis Vrije Universiteit Brussel

**Principal Investigator**

Jacques De Grève MD PhD

Dienst Medische Oncologie - Oncologisch Centrum

Academisch Ziekenhuis Vrije Universiteit Brussel

**Study Coordinators**

Jan Van Meerbeeck, MD PhD

Dienst Pneumologische Oncologie

Universitair Ziekenhuis Gent

Johan Vansteenkiste, MD, PhD

Dienst Pneumologische Oncologie

Universitair Ziekenhuis Leuven

Denis Schallier, MD, PhD

Dienst Medische Oncologie - Oncologisch Centrum

Academisch Ziekenhuis Vrije Universiteit Brussel

**Writing committee**

Jacques De Grève, Jan Van Meerbeeck, Johan Vansteenkiste, Denis Schallier

PET: Sigrid Stroobants and Hendrik Everaert

**Supported by a grant from the Stichting Tegen Kanker & unrestricted grant from Hoffman-La Roche**

| **Confidentiality statement**: This document contains confidential information that must not be disclosed to anyone other than the participating investigators and their staff involved in the conduct of the trial, the participating patients and members of the Institutional Review Board and other authorized agencies. This information cannot be used for any purpose other than the evaluation or conduct of the clinical investigation without the prior written consent of the principal investigator. |
| --- |

**Participating centres**

AZ-VUB: Prof. Dr. Jacques De Grève, dr. Denis Schallier

UZ Gent: prof. Dr. Jan Van Meerbeeck

UZ Antwerpen: prof. Dr. Paul Germonprez

CH Notre-dame Charleroi: Dr. J. L. Canon

UC Louvain: prof. Yves Humblet

Hôpital Erasme ULB: dr. Fabrice Branle

UZ Leuven: prof. Johan Van Steenkiste

AZ Middelheim: Dr. Danny Galdermans

CHU Luxemburg: dr. Guy Berchem

CHR de la Citadelle, Liège: dr. L. Bosquée

Institut Bordet: dr. A.P. Meert

CHC Liège: dr. Claude. Focan

KLINA:

St. Nicolaushospital Eupen: dr. Peter Jousten

Hôpital St. Pierre, Ottignies: dr. L. Duck

*Centrum van Glorieux zie mails*

Additional participating centres can be added through an amendment.

# Table of contents

[1 Table of contents 4](#__RefHeading___Toc116643665)

[2 Abbreviations 6](#__RefHeading___Toc116643666)

[3 Protocol synopsis 7](#__RefHeading___Toc116643667)

[4 Introduction 9](#__RefHeading___Toc116643668)

[5 Rational and aim of the study 14](#__RefHeading___Toc116643669)

[6 Study objectives and endpoints 14](#__RefHeading___Toc116643670)

[6.1 Primary objective and primary endpoint 14](#__RefHeading___Toc116643671)

[6.2 Secondary objectives and secondary endpoints 14](#__RefHeading___Toc116643672)

[7 Patient selection criteria 15](#__RefHeading___Toc116643673)

[7.1 Criteria for inclusion in the study 15](#__RefHeading___Toc116643674)

[7.2 Criteria for exclusion 16](#__RefHeading___Toc116643675)

[7.3 Concomitant Medication 18](#__RefHeading___Toc116643676)

[8 Study design and conduct. 19](#__RefHeading___Toc116643677)

[9 Response Assessments 20](#__RefHeading___Toc116643678)

[9.1 Radiological assessments. 20](#__RefHeading___Toc116643679)

[9.2 Metabolic assessment. 22](#__RefHeading___Toc116643680)

[9.3 Radiological response definitions 23](#__RefHeading___Toc116643681)

[9.4 Duration of response and survival 24](#__RefHeading___Toc116643682)

[9.5 Post-Treatment Assessments 25](#__RefHeading___Toc116643683)

[9.6 Quality of life 25](#__RefHeading___Toc116643684)

[10 Safety assessment 25](#__RefHeading___Toc116643685)

[11 Study Design and Dosing Regimen 26](#__RefHeading___Toc116643686)

[11.1 Study Design 26](#__RefHeading___Toc116643687)

[11.2 Dosing Regimen 26](#__RefHeading___Toc116643688)

[11.3 Study Duration 26](#__RefHeading___Toc116643689)

[11.4 Administration of erlotinib 27](#__RefHeading___Toc116643690)

[11.5 Description and handling of erlotinib 27](#__RefHeading___Toc116643691)

[11.6 Drug Accountability 27](#__RefHeading___Toc116643692)

[12 Safety Issues 28](#__RefHeading___Toc116643693)

[12.1 Serious Adverse Events 28](#__RefHeading___Toc116643694)

[12.2 Handling of Serious Adverse Events 29](#__RefHeading___Toc116643695)

[12.3 Expected Adverse Event 31](#__RefHeading___Toc116643696)

[12.4 Pregnancy 31](#__RefHeading___Toc116643697)

[12.5 Dose Modifications for Toxicity 31](#__RefHeading___Toc116643698)

[12.6 Period of observation for (S)AE 34](#__RefHeading___Toc116643699)

[12.7 Supportive care measures 34](#__RefHeading___Toc116643700)

[12.8 Criteria for Premature Withdrawal 35](#__RefHeading___Toc116643701)

[12.9 Pre-treatment & treatment evaluations formalities 35](#__RefHeading___Toc116643702)

[13 Tumor response evaluation formalities 39](#__RefHeading___Toc116643703)

[14 Patient study withdrawal formalities 39](#__RefHeading___Toc116643704)

[15 Statistical considerations 40](#__RefHeading___Toc116643705)

[16 Translational aspect 41](#__RefHeading___Toc116643706)

[16.1 Mutation analysis 41](#__RefHeading___Toc116643707)

[16.2 Additional biomarkers 43](#__RefHeading___Toc116643708)

[17 Documentation and use of study data 43](#__RefHeading___Toc116643709)

[17.1 Documentation of study data 43](#__RefHeading___Toc116643710)

[17.2 Intellectual property, authors rights and publications 44](#__RefHeading___Toc116643711)

[18 Ethical and legal aspects 45](#__RefHeading___Toc116643712)

[18.1 Good clinical practice (GCP) 45](#__RefHeading___Toc116643713)

[18.2 Patient study-files 45](#__RefHeading___Toc116643714)

[18.3 Patient Informed Consent 45](#__RefHeading___Toc116643715)

[18.4 Ethical Committee/Institutional Review Board 45](#__RefHeading___Toc116643716)

[18.5 Ethical Considerations 46](#__RefHeading___Toc116643717)

[18.6 Insurance 46](#__RefHeading___Toc116643718)

[18.7 Modification of the protocol 46](#__RefHeading___Toc116643719)

[19 Study monitoring 46](#__RefHeading___Toc116643720)

[20 Appendices 47](#__RefHeading___Toc116643721)

[19 Appendix 1 SAE reporting form 48](#__RefHeading___Toc116643722)

[19 Appendix 2 Informed Consent 49](#__RefHeading___Toc116643723)

[19 Appendix 3 WHO - KPS 56](#__RefHeading___Toc116643724)

[19 Appendix 4 CTC AE 3.0 57](#__RefHeading___Toc116643725)

[19 Appendix 5 Imaging Specifications 58](#__RefHeading___Toc116643726)

[20 References 60](#__RefHeading___Toc116643727)

# Abbreviations

| EGFR  TK | Epidermal Growth Factor Receptor  Tyrosine kinase |
| --- | --- |
| TKD  TKI  NSCLC | Tyrosine kinase domain  Tyrosine kinase inhibitor  Non small cell lung cancer |
| ALT | Alanine aminotransferase |
| AP | Alkaline phosphatase |
| AST | Aspartate aminotransferase |
| CR | Complete response |
| GGT | Gamma-glutamyltransferase |
| HIV | Human immunodeficiency virus |
| IV | Intravenous |
| LDH | Lactate dehydrogenase |
| NA | Not available |
| NE | Not evaluable |
| OR | Objective response according to RECIST criteria |
| PD  CR | Progressive disease  Complete response |
| PR | Partial response |
| SD | Stable disease |
| TTP | Time to progression |
| OS | Overall survival |

# Protocol synopsis

| **Title**  PROSPECTIVE EVALUATION OF SMALL MOLECULE EGFR-1 TYROSINE KINASE INHIBITION AS A FIRST-LINE TREATMENT IN PATIENTS WITH ADVANCED NON-SMALL CELL LUNG CANCER (NSCLC) HARBOURING A MUTANT EGFR GENE |
| --- |
| **Sponsor / Study Coordination**  Dienst Medische Oncologie - Oncologisch Centrum  Academisch Ziekenhuis Vrije Universiteit Brussel |
| **Study hypothesis**  Erlotinib is a safe and effective first-line treatment for NSCLC harboring a mutant EGFR |
| **Study design**  One arm, multicenter investigator initiated phase II study. |
| **Primary objective**  To investigate the efficacy and feasibility of using a small molecule EGFR inhibition in the first-line treatment of non-small cell lung cancer carrying (a) EGFR kinase domain mutation(s) not amenable to curative treatment. |
| **Primary endpoint**  Progression free survival time. |
| **Secondary objectives**  Objective response rate according to the RECIST criteria, metabolic response rate Feasibility, toxicity, duration of response and survival (TTP and OS from the date of study entry). |
| **Secondary endpoints**   - Toxicity according to the NCI-CTCAEv3 criteria. - Feasibility (reported descriptively). - Duration of response - Overall survival (from the initiation till PD). |
| **Patient selection criteria**   - - **Identification of an EGFR TKD mutation in the tumor material of the patient.** - No previous chemotherapy for metastatic disease. - Patients in whom any other anticancer treatment is needed after inclusion in the study will be considered to have met the primary endpoint. - Standard phase II recruitment criteria. |

| **Treatment plan and special procedures**  Patients will remain in the study under continuous treatment with erlotinib as long as they remain progression free, receive no other secondary anticancer treatment and remain free from unacceptable toxicity. |
| --- |
| **Study treatment duration, discontinuation and study withdrawal**   - At any time during study, progression of disease, unacceptable toxicity or patient refusal will result in study treatment discontinuation. - Patients that stop study treatment for other reasons than progressive disease must be followed by serial assessment of their response status as provided by the protocol |
| **Statistical considerations**  **Design**: phase II  **Nr of patients**: based on results obtained with chemotherapy a progression free survival of >= 60% could be expected at 3 months. The treatment will be considered for further development if a PFS > 70% can be obtained at 3 months. The treatment will be considered inappropriate as a first line if PFS < 50% at 3 months. Therefore a maximum of 40 patients will be included plus the number needed to replace patients that will drop out for reasons other than disease progression and patients not meeting eligibility criteria. |

# Introduction

Patients with stage IV NSCLC and some patients with advanced locoregional disease (stage III) are in general incurable and have a low probability for long-term survival.

Current systemic treatment in good PS patients (PS 0-1) consists of a cisplatin doublet (e.g. cisplatin plus a second drug: vinorelbine, gemcitabine, paclitaxel, docetaxel). The median and one year survival obtained with this treatment ranges between 8-10 months and 25 - 35 % respectively (1,2). Progression-free survival is a median of 5 month or less in randomized studies (3). Response rates obtained are less than 25% in metastatic disease and up to 75% in advanced locoregional disease.

Receptor tyrosine kinase inhibitors (RTKI's) are active drugs in patients with NSCLC pre-treated with cisplatin and/or docetaxel containing chemotherapy (2,4). Recently it has been shown that the EGFR1 kinase inhibitor erlotinib added to best supportive care (BSC) prolonged survival compared to BSC alone in patients with advanced NSCLC failing 1^st^ or 2^nd^ line chemotherapy (5).

A small number of preliminary reports have indicated that the objective response rate with these RTKI's as first line treatment in some patient populations with advanced NSCLC could be around 20 % (6), The objective (mainly partial) response in the phase II studies with pre-treated patients ranges from 10 - 15 % according to the level of pre-treatment but the control of the disease (stable disease) and improvement of symptoms without demonstrated objective response has been reported to be as high as 40 to 50 % (2,4). The addition of RTKI's or placebo to the current standard doublets (e.g. cisplatin, gemcitabine or taxol/carboplatin) has not been shown to impact on response rate, time to treatment failure or survival in large phase III randomised trials in advanced NSCLC patients not selected for EGFR expression (7,8).

Recently, mutations in the intracellular EGFR kinase domain that increase sensitivity of the receptor to RTKI's have been discovered (9,10). These studies suggest that the response/resistance to treatment could be strongly correlated to the presence/absence of such mutations. It is presently unclear whether patients who achieve stable disease under RTKI treatment do have receptor mutations, of which nature such mutations could be or what other biological pathways modulate the responsiveness/resistance.

Mutations have been observed in about 10 % or less of the tumours examined.

Most mutations in the EGFR kinase domain have been found in adenocarcinoma of non-smokers or minimal smokers.

Recent data have established that an EGFR mutation can be found in 30% of adenocarcinoma of the lung if the smoking history is maximally 15 years. The probability of finding a mutation is approximately 50% in never-smokers with adenocarcinoma (11).

In contrast to the rather low objective response rate, the clinical benefit rate of treatment with RTKI is around 40 % in a general population of non-small lung cancer (9,10), more than can be accounted for by mutational analysis. EGFR gene amplification might be another determinant for sensitivity, but the biological factors leading to disease stabilisation in some patients with non-activated EGFR are presently unknown.

Several humanized monoclonal antibodies (huMoAb) that can inhibit the EGFR-1 receptor have been developed as well. Of these, cetuximab (Erbitux®) has been developed most extensively, especially in head and neck (12) and colorectal cancer (13,14), but other, fully humanized antibodies (huMoAb) (ABX-EGF, EMD 72000) are under development.

Cetuximab has been assessed in NSCLC, especially in combination with first-line and second-line chemotherapy (15), but no data are currently available with respect to single agent activity.

The two types of currently available drugs targeting the EGFR-1, the RTKI's such as gefitinib and erlotinib and huMoAb such as cetuximab and ABX-EGF, are generally well tolerated and devoid of significant grade 3-4 toxicity.

While there are strong indications that the major responses obtained with RTKI’s could strongly correlate with the presence of tyrosine kinase domain mutations in addition to the known mechanism of action of these drugs (16,17), this is at present yet not so clear for the monoclonal antibodies directed against the extracellular domain of the receptor.

Some recent data suggest that EGFR gene amplification might also correlate with response to TKI (18).

Patients with Stage IV and some advanced stage III NSCLC are at present incurable and first-line chemotherapy has had only a palliative and a life prolonging effect at a cost of variable toxicity.

On the other hand the response rate to TKI in patients with a NSCLC carrying a mutant receptor is probably high as can be inferred from the comparison of the prevalence of such mutations and the response rate to these agents in an unselected population (16).

It seems therefore reasonable to explore in a first-line setting the potential of EGFR-1 targeted therapy, as these drugs have a very favourable toxicity profile and might induce prolonged palliation.

There are currently no data that would suggest that delaying chemotherapy in this patient population in well controlled conditions and for a limited time period (sufficient to evaluate the efficacy of more novel first-line treatments) might affect the efficacy of subsequent chemotherapy. There are also no data that suggest that prior administration of drugs targeted at the EGFR-1 will induce resistance to subsequent chemotherapy. The distinct respective mechanisms of action of these drugs and chemotherapy also do not suggest this to be likely.

In other diseases such as prostate cancer and oestrogen receptor-positive breast cancer, the availability of hormonal treatments permits prolonged and at times long-term disease control with limited discomfort. It is possible that a similar scenario could be obtained with an anti-EGFR treatment in NSCLC carrying a mutant EGFR.

Therefore it seems justified to expand previous second-line phase II experience with anti-EGFR strategies to patients with incurable stage III-IV NSCLC without prior therapy and a mutant EGFR. The informed consent towards these patients before entry in the study will clearly state that until now the first-line standard of care has been chemotherapy, which leads to a small overall survival benefit compared to the supportive treatment only and that participation in the current study will delay this treatment within a tightly controlled setting.

In the current trial the RTKI erlotinib will be used. Prior experience with this drug has been obtained in phase II and III studies. In the phase II setting, in an unselected pretreated population, a response rate of 12.3% and a median survival of 8.4 months could be obtained (19). In phase III, placebo-controlled studies, combination of erlotinib with concurrent chemotherapy does not provide a survival advantage (gemcitabine and cisplatin in TALENT (20) and paclitaxel and carboplatin in TRIBUTE (21). Several hypotheses can explain these results: lack of selection of patients; antagonism between cytostatic and cytotoxic agents (negative interaction with chemotherapy when given concurrently); chemotherapy and EGFR inhibitors target the same cell population since chemotherapy directly or indirectly affects EGFR function/expression and thereby reducing the effects of EGFR inhibitors. These results are in contrast to the significant survival benefit seen in the phase III randomized placebo-controlled study in NSCLC following failure of 1st or 2nd line chemotherapy (BR21). The response rate was 9% in the erlotinib treated patients, with significant increase in progression free survival (from 8 to 9,7 wks) and increased survival (from 4.7 mth to 6.7 mth, p< 0.001). In that study the biological parameters that correlate with response (mutation status, EGFR gene amplification and EGFR immunohistochemistry) are still under study.

Survival benefit correlated best with the presence of EGFR expression by immunohistochemistry and EGFR gene polysomy (22).

# Rational and aim of the study

Current chemotherapy for advanced non-small cell lung cancer, not amenable for curative local treatment (surgery or chemoradiotherapy), has a modest life-prolonging effect and can improve quality of life. There is however no potential for long-term cure for these patients.

Chemotherapy also produces variable and often significant toxicity.

Current retrospective evidence suggests that significant clinical responses can be obtained when patients whose cancer cells have an EGFR TKD mutation are treated with an EGFR TKI.

The ease of administration and toxicity profile of TKI compare favourably with that of chemotherapy, even single agents such as for example gemcitabine

The present study will establish the clinical benefit rate of TKI as a first line treatment in patients with EGFR mutations and thus estimate the proportion of patients who might benefit for a prolonged period from a treatment with a modest toxicity profile.

# Study objectives and endpoints

## Primary objective and primary endpoint

Establish clinical benefit (progression free survival) of first line RTKI in patients with stage IV and stage IIIB NSCLC not eligible for curative-intent treatment (chemo-radiotherapy) carrying a mutant EGFR-1.

## Secondary objectives and secondary endpoints

- - Determine response rate (OR and stable disease) and duration under erlotinib treatment.
- Determine the effect on Quality of Life (QOL) of first-line anti-EGFR-1 treatment.
- Determine the value of positron emission tomography (PET)-scan as an early predictor of response and clinical benefit.
- Overall survival from the time of study entry to the date of death or date of last follow-up.
- Determine biological correlates for response/resistance in tumour tissues.

# Patient selection criteria

## Criteria for inclusion in the study

- Histological or cytological documented diagnosis of inoperable, locally advanced, recurrent or metastatic (Stage IIIB or Stage IV) adenocarcinoma of the lung in a patient with a smoking history of < 15 years and quit smoking > 1 year before diagnosis.
- Patients must have evidence of disease but measurable disease is not mandatory.
- 18 years of age or older.
- ECOG performance status of 0 – 3.
- Patients not eligible for standard curative-intent treatment with surgery or chemo-radiotherapy.
- Life expectancy ≥ 3 months.
- Prior therapy for NSCLC allowed for primary disease: surgery and radiotherapy and adjuvant or proto-adjuvant chemotherapy completed > 6 months before inclusion
- Adequate bone marrow, hepatic and renal function:
  - Granulocyte count > 1.5 x 10^9^/L and platelet count > 100 x 10^9^/L
  - Serum bilirubin must be < 1.5 upper limit of normal (ULN).
  - If alkaline phosphatase is > 2.5 x ULN, SGOT (AST) and SGPT (ALT) must be < 1.5 x ULN.
  - Serum creatinine < 1.5 ULN or creatinine clearance > 60 ml/min.
- Ability for giving informed consent for participating in the study and filling out FACT-L quality of life scales.
- Able to comply with study and follow-up procedures.
- Availability of tumour biopsy sample (fixed in formalin and, if possible, also snap frozen tumour sample). If frozen samples are available, these will be collected by central data management.
- Informed Consent for performing mutation analysis and subsequent biomarker analysis.
- Separate signed Informed Consent for participation in the treatment phase of the study.
- Ability to take oral medication.
- For all females of childbearing potential a negative pregnancy test must be obtained within 48 hours before starting therapy.
- Patients with reproductive potential must use effective contraception.

## Criteria for exclusion

- Patients with previously untreated disease for whom urgent chemotherapy or radiotherapy is deemed necessary (e.g. rapidly progressive disease).
- Absence of tumour biopsy material available for mutation analysis.
- Current symptomatic central nervous disorder, brain or leptomeningeal metastasis. Previously diagnosed and treated CNS metastases or spinal cord compression with evidence of stable disease (clinically stable imaging) for at least 2 months is permitted.
- Pre-existing symptomatic interstitial lung disease, not related to the current malignancy.
- Patients with a history of other malignancies, except patients with basal cell carcinoma of the skin or in situ carcinoma of the cervix with a disease free interval of ≥ 5 years. Patients with a prior history of other good prognosis malignancies more than 5 years since end of treatment and in un-maintained complete remission also can be considered for inclusion after discussion with the Principal Investigator
- Prior therapy with systemic anti-tumour therapy with HER1/EGFR inhibitors (small molecule or monoclonal antibody).
- Prior chemotherapy for metastatic disease.
- Concomitant treatment with corticosteroids, except chronic treatment lasting more than one month, at low dose (≤ 20mg of methylprednisolone or equivalent per day).
- Significant malabsorption syndrome or disease affecting the gastrointestinal tract function
- Pregnant or breast-feeding women; for women in reproductive condition, a negative pregnancy test is required.
- Male or female patients with reproductive potential who do not use an approved contraceptive method during and for 3 months after the end of the treatment.
- Concomitant food or drug intake which potentially impairs absorption and metabolisation of RTKI's.
- Psychological, familial, sociological or geographical conditions potentially compromising compliance with the study protocol and follow up schedule.
- Participation in another clinical trial with any investigational drug within 30 days prior to study screening.
- Any unstable systemic disease (including active infection, grade 4 hypertension, unstable angina, congestive heart failure, hepatic, renal or metabolic disease).
- Any significant ophthalmological abnormality, especially severe dry eye syndrome, keratoconjunctivitis sicca, Sjögren syndrome, severe exposure keratitis or any other disorder likely to increase the risk of corneal epithelial lesions. The use of contact lenses is not recommended during the study. The decision to continue to wear contact lenses should be discussed with the patient’s treating oncologist and ophthalmologist.
- Nursing mothers.

## Concomitant Medication

- Patients who have evidence of disease progression requiring radiation therapy should discontinue protocol treatment.
- Patients should receive full supportive care per institutional standards.
- Concomitant biphosphonate use to control bone pain is permitted.
- Patients with dry eyes (an abnormal Schirmer’s test results on baseline eye exam) should be advised to use an ocular lubricant.
- Concomitant treatment with warfarin or coumarin-derivative is permitted provided increased vigilance occurs with respect to monitoring INR.
- Administration of any other anti-cancer therapy (cytotoxic, biological/immunotherapy or full dose radiotherapy) is not permitted until after disease progression is documented. After permanently discontinuing the protocol treatment, patients may be treated at the investigators discretion.
- Patients who receive protocol treatment should not receive ANY other (non-anticancer) investigational drugs until after the post-treatment assessment (at least 30 days after the final dose of protocol treatment).
- Patients who continue to wear contact lenses may have an increased risk of ocular adverse events. The decision to continue to wear contact lenses should be discussed with the patient’s treating oncologist and ophthalmologist.

**Potential for Drug Interactions.**

Substances that are potent inhibitors of CYP3A4 activity (eg, ketoconazole) decrease erlotinib metabolism and increase erlotinib plasma concentrations. This increase may be clinically relevant as adverse experiences are related to dose and exposure; therefore, caution should be used when administering CYP3A4 inhibitors with erlotinib.

Substances that are potent inducers of CYP3A4 activity (eg, rifampin, phenytoin) increase erlotinib metabolism and significantly decrease plasma concentrations. This decrease in exposure may be clinically relevant, as preclinical studies suggest that higher concentrations are more efficacious in in vivo animal tumour models. However, the relationship between exposure and efficacy in cancer patients has not been adequately studied. See Appendix 4: CYP3A4 Inhibitors.

International Normalized Ratio (INR) elevations and/or bleeding events have been reported in some cancer patients taking warfarin while on erlotinib. Patients taking warfarin or other coumarin-derivative anticoagulants should be monitored regularly for changes in prothrombin time or INR.

# Study design and conduct.

Patients diagnosed with advanced non-small cell lung cancer and meeting the selection criteria can be considered for study inclusion. Patients concomitantly receive the Informed Consent form for mutation analysis of their tumor material and the Informed Consent for the treatment phase of the study. The Informed Consent for the mutation analysis includes the possibility for a repeat biopsy if the available material is insufficient and a biopsy is possible and safe. It also includes the possibility of performing a second biopsy (if possible and safe) upon progression under protocol treatment for study of secondary resistance mechanism. After obtaining both consents, the baseline examinations needed for the study can be performed in parallel to the mutation analysis, the result of which should be available within 2 weeks. Patients will be included in the study after filing of the inclusion form with the central data management and principle investigator. Eligibility for subsequent protocol treatment depends on the identification of a kinase domain mutation in the TK domain of the EGFR.

The maximal interval allowed between baseline exams and initiation of study is 3 weeks.

After documentation of unequivocal objective progression the patients will go off study. If the patient leaves the study because of unacceptable toxicity or any other reason without progressive disease, the assessments for determination of response duration will continue until disease progression is documented.

### **Summary of study flow.**

### Tumour biopsy for diagnosis and EGFR mutation analysis.

### In view of the time needed to perform the mutation analysis, it is preferable (but not mandatory) to obtain this sample at the time of the initial diagnosis.

### Separate Informed Consents for mutation analysis and participation in the study.

### Baseline evaluation, during which the mutation screen is being performed.

### Evaluation of eligibility.

### Inclusion and treatment.

### Response assessments (imaging and physical) and ancillary parameters at 6, 12, 18 and 24 weeks. Thereafter every 12 weeks until disease progression (RECIST definition). A first metabolic response assessment will be at 2 weeks and subsequently synchronized with the radiological evaluations. A concomitant CT-scan at 2 weeks is recommended.

### Patients remain in study until disease progression/unacceptable toxicity.

# Response Assessments

## Radiological assessments.

Up to a maximum of five measurable lesions for each site are identified as target lesions and measured at baseline. The sum of the longest diameter for all target lesions will be calculated and reported as the baseline sum longest diameter, which will be the reference to evaluate the tumor response to treatment. The same method of assessment and the same technique should be used to characterize each identified and reported lesion at baseline and during follow-up.

Other lesions should be identified as non-target lesions and should also be recorded, but not measured, at baseline. They should be followed as “present” or “absent”.

Patients without measurable lesions will be scored for disease progression, also according to Recist criteria.

RECIST (Uni-dimensional Tumour Measurement) will be used to evaluate best response during the treatment by the local investigator (23).

Confirmation of responses (CR, PR) has to be performed at least 4 weeks after initial documentation, in practice it will normally coincide with the next scheduled 6-weekly evaluation.

Evidence of progression will be reviewed by the local investigator and the principal investigator before ending erlotinib treatment. All responses will also be reviewed at the end of the study centrally in collaboration with the writing committee of the study.

Each subject will be assigned a best objective response as per investigators decision (according to RECIST criteria, see Appendix 2). This is defined as the best response recorded from the start of treatment until disease progression/recurrence. Objective response will be summarized in a descriptive manner.

## Metabolic assessment.

The PET-scan imaging with FDG will be performed at baseline, at day 14 and at 6 weeks and 12 weeks. The evaluation of the PET-scan at 2 weeks is essential for assessing the predictive value of PET imaging for radiological response determination. The PET-scan will in no instance be used to make decisions about continuation or discontinuation of the experimental treatment, which only relays on the radiological and clinical assessments. The continuation of repeat PET-scan at week 12 and subsequent assessments will be in consultation with Sigrid Stroobants, Hendrik Everaert and the Principal Investigator on a case-by-case basis. The PET acquisition protocol should be as simple as possible (WB-FDG with AC). The protocol should be according to local standards but standardized: For each patient the same camera should be used, similar at the G-dose, glucose measurement and same acquisition and reconstruction protocol. The scan should start 60 to 90 minutes post injection.

PET data analysis. Quantitative PET analysis

- SUV max, glucose correction + largest metabolic lesion diameter. Local reading and recorded in CRF. EORTC PET criteria will be applied for metabolic response determination: up to 5 target lesions (diameter > 2 cm). The target lesions for PET and radiological imaging can be different. Metabolic complete response is complete normalisation of FDG uptake, metabolic partial remission is 25% decrease, SUV max. in all target lesions, metabolic progressive disease is 25% increase, SUV max. in one target lesion or 25% increase in lesion diameter if one target or appearance of no lesions. Metabolic stable disease is all other.

## Radiological response definitions

### Target lesions

- Complete response (CR): disappearance of all target lesions.
- Partial response (PR): At least a 30% decrease in the sum of the longest diameter of target lesions taking as reference the baseline sum.
- Stable disease (SD): Neither sufficient shrinkage to qualify for partial response nor sufficient increase to qualify for progressive disease taking as reference the smallest sum of the longest diameter of target lesions since the treatment started.
- Progressive disease (PD): At least a 20% increase in the sum of the longest diameter of target lesions taking as reference the smallest sum of diameter for all target lesions recorded since the treatment started.

### Evaluation of non-target lesions and new lesions

- Complete response (CR): disappearance of all non-target lesions.
- Incomplete response/stable disease: the persistence of one or more non-target lesion(s)
- Progressive disease (PD): unequivocal progression of existing non-target lesions and/or the appearance of one or more new lesions.

### Evaluation of best overall response

The best overall response is the best response recorded since the start of treatment until disease progression or recurrence, taking as reference for progressive disease the smallest measurements recorded since the treatment started. Table 1 below provides overall responses for all possible combinations of tumor responses in target and non-target lesions, with or without the appearance of new lesions.

Table 1

Target lesions Non-target lesions New lesions Overall response

CR CR No CR

CR Incomplete response/SD No PR

PR Non-PD No PR

SD Non-PD No SD

PD Any Yes or no PD

Any PD Yes or no PD

Any Any Yes PD

### Response confirmation

CR, PR and SD must be confirmed by a complete tumor staging performed no less than 4 weeks (usually at the next scheduled 6 week interval evaluation) after the criteria for response are first met (normally at the next preplanned evaluations).

## Duration of response and survival

The duration of response (CR or PR) will be calculated from the time point a PR or CR has been documented until the documentation of progression.

The time to progression (TTP) will be calculated from the date of recruitment to the study until the documentation of progression, the latest date of follow up for patients who are lost to follow-up or the date of death.

Overall survival will be calculated from the date of recruitment to the study until the date of death or latest date of follow up for patients who are lost to follow-up.

### Time to Progression

The Time to Progression is defined as the time from start of treatment to the date of the first documented progression (according to RECIST criteria) or the date of death for any reason in the absence of PD. Patients who have not died or progressed at the time of the final analysis will be censored at the date of last contact.

### Survival Assessment

Overall survival will be determined from the date of start of treatment to the date of death irrespective of the cause of death. At the end of the study a survival follow up report for each patient is required. Patients who have not died at the time of the final analysis will be censored at the date of last contact.

Time to progression and survival will be summarized using Kaplan-Meier methodology.

## Post-Treatment Assessments

After discontinuation of erlotinib treatment, patients will be followed for response duration, if still progression free and until disease progression according to protocol schedule. Any serious erlotinib related toxicity must continue to be reported until resolution or until no further change is expected.

## Quality of life

Karnofsky performance score, weight and FACT-L questionnaire will be assessed at baseline and at disease evaluation time points.

# Safety assessment

At 2 weeks after treatment initiation and subsequently at the disease evaluation time points.

- Physical examination, vital signs, weight, PS
- Adverse events NCI CTC version 3.0 grades
- Haematology, biochemistry including renal and liver function tests (ureum, creatinine, SGOT, SGPT, bilirubine levels).

# Study Design and Dosing Regimen

## Study Design

This is an open label non-randomized multicentre phase II trial in patients with advanced (inoperable stage III or IV) NSCLC (adenocarcinoma) who have tumour material available for EGFR mutation analysis and in whom a kinase domain mutation has been identified.

## Dosing Regimen

**The first dose of erlotinib must be given within 1 week after establishment of a gene mutation.**

The mutation status will be communicated via the central data management to the participating investigators. All enrolled patients will receive erlotinib (150 mg/day). No dose escalation of erlotinib is permitted. Erlotinib will be dose reduced for toxicities as detailed in section 12. Toxicity will be graded according to the NCI CTCAE v3.0. Treatment may be delayed for ≤ 2 weeks to allow recovery from toxicity.

Patients will be treated until progression of disease or unacceptable toxicity.

## Study Duration

The recruitment will continue until the number of patients foreseen has been accrued. Patients will remain on treatment until withdrawal due to toxicity, disease progression or withdrawal due to any other reason. The study will be terminated after every living patient has had a follow up of at least 6 months after stopping erlotinib, or when all patients have died.

## Administration of erlotinib

Erlotinib will be given on an outpatient basis at a fixed dose of 150 mg as a single daily oral dose. Dosage is not based upon body weight or body surface area. Prescribed daily dose is to be taken preferably in the morning, with up to 200 mL of water. Drug should be taken at least 1 hour before or 2 hours after the ingestion of any food or other medications, including grapefruit juice, vitamins and iron supplements.

Missed daily doses should be skipped. Doses should be taken at the same time each day. If the patient vomits after taking the tablets, the dose is replaced only if the tablets can actually be seen and counted. If a patient misses a dose normally taken in the morning, s/he may take the dose any time during that same day. However, the missed dose should not be taken on a subsequent day.

## Description and handling of erlotinib

For a description of handling, drug formulation, packaging and storage see the Tarceva^TM^ Investigators Brochure, which will be provided by F HOFFMANN-LA ROCHE.

## Drug Accountability

Free drug is made available for this study by and directly delivered to the investigators, after confirmation of inclusion by the central data office.

Drug accountability logs record quantities are received directly from F HOFFMANN-LA ROCHE.

Quantities dispensed to patients, including lot number, date dispensed, patient identifier number, patient initials, protocol number, dose, quantity returned, batch number, expiry date and the initials of the person dispensing the medication has to be maintained per institutional standards.

# Safety Issues

Safety will be evaluated by collection of all serious adverse events and adverse events leading to premature withdrawal. In addition, unexpected erlotinib related AEs and erlotinib related rash must be reported. An unexpected AE is one which specificity or severity is not consistent with the current version of the erlotinib Investigator’s Brochure.

Severity should be recorded and graded according to the NCI CTCAE, v3.0. The CTC manual can be found at the following website

<http://ctep.cancer.gov/reporting/ctc.html>

A drug-related AE is defined as, a noxious and unintended response to any dose of a drug product for which there is a reasonable possibility that the product caused the response.

This criterion, in addition to good clinical judgment, should be used as a guide for determining the causal assessment.

## Serious Adverse Events

A serious adverse event is any experience that suggests a significant hazard, contraindication, side effect or precaution. It is any adverse event that at any dose fulfils at least one of the following criteria:

- is fatal (results in death) (unless clearly due to disease progression) (note: death is an outcome, not an event)
- is life-threatening (note: the term “life-threatening” refers to an event in which the patient was at risk of death at the time of the event; it does not refer to an event which could hypothetically have caused death had it been more severe)
- required patient hospitalization or prolongation of existing hospitalization (note: “inpatient hospitalization” refers to an unplanned, overnight hospitalization)
- results in persistent or significant disability/incapacity
- is a congenital anomaly/birth defect
- is medically significant or requires intervention to prevent one or other of the outcomes listed above

Medical and scientific judgment should be exercised in deciding whether expedited reporting to the Coordinator is appropriate in other situations, such as important medical events that may not be immediately life-threatening or result in death or hospitalization but may jeopardize the patient or may require intervention to prevent one of the outcomes listed in the definitions above. These situations should also usually be considered serious. Examples of such events are intensive treatment in an emergency room or at home for allergic bronchospasm; blood dyscrasias or convulsions that do not result in hospitalization; or development of drug dependency or drug abuse.

## Handling of Serious Adverse Events

Any adverse event that is considered SERIOUS must be reported within one working day by the investigator to the central data office, which will also inform the Roche. Roche is still responsible for reporting SAEs to the authorities, in any countries where there are ongoing trials involving erlotinib (erlotinib)

An unexpected adverse event is one, of which the nature or severity is not consistent with the safety information in the investigator’s brochure.

Investigators must assess the causal relationship between the event and the study drug. There are only 2 possibilities:

- “NO” no reasonable suspicion of a causal relationship
- “YES” reasonable suspicion of a causal relationship

The term severe is a measure of intensity, thus a severe adverse event is not necessarily serious. For example, nausea of several hours' duration may be rated as severe, but may not be serious.

A serious adverse event occurring during the study, or which comes to the attention of the investigator within 28 days after stopping the treatment, whether considered treatment-related or not, must be reported. In addition, a serious adverse event that occurs after this time, if considered related to the study medication, should be reported.

On request from the sponsor such preliminary reports will be followed by detailed descriptions later, which will include copies of hospital case reports, autopsy reports and other documents.

The Serious Adverse Event should be reported on the SAE form which can be found as an attachment to the CRF and should be faxed to the central data office contact specified below.

**<Datamanagement>**

**<Mr. Nicolas Fontaine>**

**<Oncologisch Centrum>**

**<AZ-VUB>**

**<Tel 32-2-4775461>**

**<Fax 32-2-4775460>**

**Contact for SAEs:**

1. The central data management office by tel.: 02/477.5461 and/or email ([Nicholas;Fontaine@az.vub.ac.be](mailto:Alex.Dewaele@az.vub.ac.be)) and/or fax 02/477.60.12
2. The local institutional ethical committee (within the first 24h of the following weekday).

The investigator must notify his/her Ethics Review Committee/Institutional Review Board of a serious adverse event in writing as soon as is practical and in accordance with international and local laws and regulations.

The investigator should report all pregnancies in the study within 24 hours to the same central datamanagement office, using the form for Serious Adverse Events. On receipt of this form, a special pregnancy follow-up form will be provided for the documentation of relevant data (ante-natal ultrasound, health of baby, etc.). This form must be returned within 24 hours of any fatal anomaly being detected, or as soon as possible after a normal birth.

## Expected Adverse Event

The most common, expected AEs for erlotinib include: rash (dermatosis), diarrhea (with or without dehydration), nausea, vomiting, fatigue, stomatitis, headache, and dry eyes. Less frequent but more severe events include infections without neutropenia, liver function test abnormality, INR elevation, GI hemorrhage, and rare reports of interstitial lung disease. For a complete list of expected AEs, please see the current version of the erlotinib Investigator’s Brochure.

Erlotinib related AEs not described in the erlotinib Investigator’s Brochure, regardless of seriousness should be reported on the Adverse Event Report Form included in the CRF.

## Pregnancy

A female subject must be instructed to stop taking the test “drug” and immediately inform the investigator if she becomes pregnant during the study. Pregnancies occurring up to 90 days after the last dose of the test “drug” must also be reported to the investigator. (add if approprThe investigator should report all pregnancies within 24 hours to the principle investigator. The investigator should counsel the subject; discuss the risks of continuing with the pregnancy and the possible effects on the foetus. Monitoring of the patient should continue until conclusion of the pregnancy.

Pregnancy occurring in the partner of a subject participating in the study should also be reported to the investigator and the sponsor. The partner should be counselled and followed as described above.

## Dose Modifications for Toxicity

Dose adjustments are to be made according to the system showing the greatest degree of toxicity (see Table 2).

The most common adverse effects of erlotinib are diarrhoea and skin rash. Other reported adverse effects include fatigue, dry skin, pruritus, anorexia, nausea, vomiting, dry mouth; dry eyes and headache.

In the event of toxicity (e.g., diarrhea, rash) that is not controlled by optimal supportive care, or not tolerated due to any reason, regardless of severity, the daily dose of erlotinib will be decreased to 100 mg/day. A second reduction to 50 mg is possible. Patients who cannot tolerate this dose reduction will be permanently discontinued from the program.

**Table 2: erlotinib Dose Level Reductions**

| **Starting Dose** | **First Reduction** | **Second Reduction** |
| --- | --- | --- |
| 150mg | 100mg | 50mg |

Within 2 weeks following a dose reduction, erlotinib related toxicity must improve by at least one NCI-CTC grade and be NCI-CTC Grade ≤ 2 (Any ocular toxicity must improve to NCI-CTC Grade ≤1), or further dose reduction by one level will be required. Dosing may be interrupted for a maximum of 2 weeks if clinically indicated and if the toxicity is not controlled by optimal supportive medication.

Once a patient has had a dose reduction for toxicity, the dose will not be re-escalated except in the case of erlotinib related rash. In the event of a rash, dose can be re-escalated when rash is ≤ Grade 2 (see table 3).

**Table 3: Dosage Modification Criteria and Guidelines for Management of Study Drug Related Toxicities**

| **Toxicity,  NCI-CTC Grade** | **Study Drug Dosage Modification** | **Guideline for Management** |
| --- | --- | --- |
| ***Diarrhea*** |  |  |
| Grade 1 | None | Consider Loperamide (4mg at first onset, followed by 2mg every 2 – 4 hours until diarrhea free for 12 hours)  Appropriate rehydration |
| Grade 2 | None | Loperamide (4mg at first onset, followed by 2mg every 2 – 4 hours until diarrhea free for 12 hours)  Appropriate rehydration |
| Grade 3 | Interrupt erlotinib | Loperamide (4mg at first onset, followed by 2mg every 2 – 4 hours until diarrhea free for 12 hours) **and** Interrupt erlotinib until resolution to Grade ≤ 1, and restart at reduced dose  Appropriate rehydration |
| Grade 4 | Discontinue Study | Loperamide (4mg at first onset, followed by 2mg every 2 – 4 hours until diarrhea free for 12 hours)  Appropriate rehydration |
| ***Rash*** |  |  |
| Grade 1 | None | Any of the following: minocycline ^a^, topical tetracycline, topical clindamycin, topical silver sulfadiazine, diphenhydramine, oral prednisone (short course) at discretion of the investigator. |
| Grade 2 | None | Manage as described above |
| Grade 3  (or intolerable Grade 2) | Dose reduction; dose can be re-escalated when rash is ≤ Grade 2 | Manage as described above |
| Grade 4 | Discontinue Study | Manage as described above |
| ^a^ Recommended dose: 200 mg po bid (loading dose) followed by 100mg po bid × 7–10 days. | | |

## Period of observation for (S)AE

The period of observation for collection of adverse events extends from the time of patient recruitment to the study until 30 days following the end of the last treatment cycle.

If the investigator detects a (serious) adverse event in a study patient after the end of the observation period, and considers the event possibly related to prior study treatment, he or she should contact the study coordinator to determine how the adverse event should be documented and reported.

Patients who require an interruption in dosing of > 2 weeks (due to a
erlotinib related adverse event not manageable with optimal supportive medication) will discontinue erlotinib treatment and be taken off study. Erlotinib re-challenge after interruption of dosing will occur only if the investigator considers it is in the best interest of the patient to re-challenge.

## Supportive care measures

### Diarrhea

Diarrhoea has been commonly observed (~ 50%) and is usually transient in nature. Previous trials have shown that the frequency and severity of diarrhoea rarely hindered administration of erlotinib and could be managed with loperamide. The recommended dose is loperamide 4 mg at first onset, followed by 2 mg q 2 – 4 hours until diarrhoea free for 12 hours.

### Rash

Skin rash or dermatosis (Grade 1 – 2) has been observed during the first several days of treatment with erlotinib in more than 50% and has been observed to diminish in severity despite continued treatment in many patients. In some patients, particularly when a superimposed infection is suspected, this rash appeared to improve with topical and oral antibiotics. Anecdotal reports of improvement have occurred with several agents. In patients with severe rash, treatment may need to be discontinued or the dose reduced (see table 11 above).

See Appendix 5 for additional guidelines on the treatment of rash.

## Criteria for Premature Withdrawal

The local investigator and the principle investigator have the right to withdraw subjects from the study in the event of intervening illness, adverse events or treatment failure after a prescribed procedure, protocol violations, cure, administrative reasons or other reasons.

If the reason for removal of a subject from the study is an adverse event or an abnormal laboratory test result, the principal specific event or test will be recorded on the Case Report Form.

## Pre-treatment & treatment evaluations formalities

The participating investigator in charge of the patient is responsible for verifying that the patient is eligible before inclusion in the study. If any of the inclusion criteria are not met and any of the exclusion criteria are present, the patient must not be enrolled. Once a patient is enrolled in the program, he/she will only be identified by his/her initials and the assigned patient number.

Page one and two of the CRF will be considered as the Inclusion Form, documenting the patient’s fulfilment of the eligibility criteria and the completion of the written informed consent. These CRF pages have to be completed by the investigator and faxed to the central data management office at **32-2-4775460.**

All sites as well as the central data office will maintain a patient log for all included patients.

### Schedule of Assessments

Table 4.

|  | **Screening/**  **Baseline**  **Maximum days prior to first treatment** | **Treatment Period**  **Timing** | **End of Study** |
| --- | --- | --- | --- |
| Informed Consent | 3 weeks | - | - |
| Demographics | 3 weeks | - | - |
| Medical history | 3 weeks | - | - |
| Physical examination | 3 weeks | Every 6 weeks | X |
| Pregnancy test^1^ | 48 hours before starting erlotinib | - | - |
| Laboratory^2^ | 2 weeks | Every 6 weeks | X |
| Tumor measurements^3^ | 2 weeks | - On day 14.  - Then every 6 weeks from treatment initiation, up to 6 months of follow-up  - Thereafter q 12 weeks | X |
| Tumor Biopsy and mutation analysis ^4^ | pretreatment |  |  |
| ECOG Performance Status | 7 days | Every 6 weeks | X |
| erlotinib administration |  | Continuously | - |
| Safety^5^ | - | Recorded throughout study | X |
| Survival | - | - | X |

1. In women of childbearing potential only
2. Hematology: Hemoglobin, WBC, granulocytes and platelet count, INR (Only for patients receiving warfarin or coumadin-derivative. To be done twice per week during for 3 weeks; then weekly or more often as clinically indicated.) Biochemistry: Alkaline phosphatase, Serum bilirubin, Serum creatinine, AST (SGOT) and ALT (SGPT). Only laboratory abnormalities linked to unexpected clinical symptoms will be documented in the CRF.
3. CT scans, MRI or X-ray as per Institutional Standards and FDG-scan (appendix 5 for CT-scan and FDG-scan specification). The first FDG scan is at day 14. It is also proposed that a CT-scan is performed at the same time point.
   For responding patients, a confirmatory evaluation should be repeated at least 4 weeks (normally at the next 6 week interval evaluation) after the initial determination of response.
4. A formaline fixed paraffin tissue block representative of the patient's primary cancer at the initial diagnosis or a pretreatment bronchoscopy biopsy or a biopsy from a metastatic site will be sent to the Laboratory of Molecular Oncology, Oncology Centre, AZ-VUB.
5. Serious Adverse Events, Adverse Events leading to premature withdrawal, unexpected erlotinib related AEs and erlotinib related rash.

### Study Assessments Recording Formalities

#### Clinical Assessments

All patients will provide written informed consent. The following will be completed according to the schedule of assessments (table 4):

- Demographics including smoking status (Never smokers with a lifetime exposure of fewer than 100 cigarettes, versus Former Smokers (with a maximum of 15 pack years): number of pack years.
- ECOG Performance Status
- Tumor measurement (CT, MRI or X-Ray AND FDG) with assessment of tumor done according to RECIST. For responding patients, a confirmatory evaluation should be repeated at least 4 weeks after the initial determination of response.
- Safety: Serious Adverse Events (SAEs, Adverse Events leading to premature withdrawal, unexpected erlotinib related AEs and erlotinib related rash)

#### Laboratory Assessments

The following will be completed according to the schedule of assessments

- For all females of childbearing potential a negative pregnancy test (urine or serum) must be obtained locally within 48 hours before starting therapy.
- Hematology
- Hemoglobin
- WBC, granulocytes and platelet count
- INR (Only for patients receiving warfarin or coumadin-derivative. To be done twice per week during 3 weeks; then weekly or more often as clinically indicated.)
- Biochemistry
- Serum bilirubin
- Serum creatinine
- Alkaline phosphatase
- AST (SGOT)
- ALT (SGPT)

#### Biopsy Collection

A formalin fixed paraffin tissue block representative of the patient's primary cancer or a metastatic site will be send to prof. dr. Peter In 't Veld, EXPA FIELT AZ-VUB, Laarbeeklaan 101, 1090 Jette, together with the "Request for EGFR mutation analysis form" and "Screening Eligibility Form".

The methodology for tissue sampling is further elaborated in chapter 15. Several alternative phone numbers are provided:

**Tel: 024763983 (Nicolas Fontaine) or 024763990 (Alex Dewaele) or 024763981 (Katrien Vandenbossche) or 024776077 (Erik Teugels) or 024776415 (Ingrid Bettens) or 024776211 (Sonia Goossens)**

# Tumor response evaluation formalities

Clinical tumor response will be evaluated according to the Response Evaluation Criteria in Solid Tumors (RECIST) guidelines (appendix 2). An electronic version of the reference publication on RECIST methodology will be provided on CD-ROM. For matters of discussion, the FAQ-web page concerning RECIST methodology at the EORTC website will be consulted ([www.eortc.be](http://www.eortc.be/)). The details of the response assessment are described in section 9.

FDG-PET and CT of disease sites (as well as other imaging exams needed to evaluate local or metastatic disease) will be used to evaluate disease status at base line, 2 weeks (FDG-PET obligatory, CT-scan optional) and weeks 6 and 12 post treatment initiation.

Then first 6 months post-treatment initiation, CT-scans will be performed every 6 weeks. Subsequent re-evaluations will be performed at least every 12 weeks until disease progression in accordance with current clinical management.

Response Rate, Metabolic Response Rate, Response: definitions in section 9.

# Patient study withdrawal formalities

Patients that participate in this study may be withdrawn from the study for the following reasons (different from disease progression or unacceptable toxicity):

- At their own request or at the request of their legally authorized representative.
- In case the investigator considers that continuation of study treatment is detrimental to the patients’ well being.
- At the specific request of the sponsor

In all cases, the reason for and date of withdrawal must be recorded in the case report form and in the patients’ medical records. The patient must be followed up to establish whether the reason was an adverse event, and, if so, this must be reported in accordance to the procedures related to (S)AE reporting.

The decision of a patient to withdrawal from the study for whatever reason will have no influence on the attitude of the investigator, the members of his team or the nursing group. The patient will continue to receive best treatment as estimated by the investigator.

# Statistical considerations

The purpose of this study is to obtain an estimate of the proportion of patients experiencing a progression free survival (PFS) at three months in order to position this type of first-line therapy for further study in comparison to the results that can be obtained with current first-line chemotherapy in advanced non-small cell lung cancer.

The premises for the sample size determination are as follows:

With current chemotherapy, progression free survival of 60% or less is obtained at 3 months follow-up^3^.

Taking into account the general better tolerability of RTKI’s it is proposed that if a PFS of > 70% can be obtained, that this treatment definitely deserves further evaluation against chemotherapy in the first-line treatment of advanced non-small cell lung cancer.

If the study results in a PFS less than 50 % at three months, single agent RTKI’s should not be further evaluated in this setting as outcomes inferior in terms of efficacy to the current standard are not acceptable.

Based on these premises, a one step Fleming design requires at least 33 patients to be included in the study (α= 10%; β = 10%).

With the provision of a margin for eligibility/evaluation issues, the prospective number of patients to be included will be 40 patients.

When, alternatively, statistics normally used for phase II design based on response, are applied to the PFS outcome proportions (freedom from progression regarded as a clinical benefit response), then, in a Simon minimax two-stage design and with α = 5%, β = 20%, for rejecting the null hypothesis (p0) of a freedom from progression rate < 60%, in a first stage, more than 8/13 patients should have a progression free survival of > 3 months. The second stage would lead to an accrual of 35 evaluable patients of whom at least 26 should be free from progression at 3 months to establish a probable freedom from progression rate of > 80% at 3 months. Again, with the provision of a margin for eligibility/evaluation issues, the prospective number of patients to be included will be 40 patients.

With an estimated maximal 10% mutation rate in the overall population of NSCLC, this cohort of patients could result from a baseline population of at least 400 patients with advanced non-small cell lung cancer.

Survival (time to progression and overall survival) will be calculated from the start of therapy until disease progression or death or last follow-up according to the Kaplan-Meier method with SPSS statistical software (release 7.5, 1996; SPSS Inc, Chicago, IL).

# Translational aspect

## Mutation analysis

Paraffin embedded and formalin fixed tissue will be collected at diagnosis with an informed consent before inclusion in the trial. If possible frozen tissues will also be collected.

Preferably percutaneous biopsies of primary and/or metastatic disease sites will be obtained. Endobronchial biopsies can also be considered for mutations analysis but the success rate might be less. If the patient was treated for locoregional disease, biopsy material from that treatment episode is also suitable for mutation analysis. Surgical specimens will increase the mutation detection rate significantly.

**Recommended fixation method:**

Solution: Neutral buffered 4% Formaldehyde

100ml 40% formal in 900ml distilled H_2_O and 4g NaH_2_PO_4_ - H_2_O + 6,5g Na_2_HPO_4_

Fixation duration: < 24 hrs, **preferably 6-8h**. For small endobronchial biopsies 2hrs is sufficient.

The presence of EGFR mutations in the tumour DNA will be determined or at least confirmed if initially determined elsewhere, in the Laboratory of Molecular Oncology (Dr. Erik Teugels) of the Department of Medical Oncology-VUB. To maximally increase the mutation rate in the samples considered for mutation analysis, the patients will be preselected on the basis of their phenotype: adenocarcinoma in never-smokers or former smokers who quit smoking > 1 year before inclusion and with less than 15 pack years of smoking.

A tissue slice with a volume of 2 mm^3^ can reliably be analysed. In a significant proportion the kinase domain mutations can also be determined in very small amount of tissues, but then the chance of finding a mutation decreases. In samples containing less than 50% tumour samples, tissue macro/microdissection will be used to enrich for malignant cells.

Other participating centres can, if they wish, have the samples analysed in their own laboratories and, upon finding a mutation, include the patient in the study. Later the mutation needs to be confirmed in the central lab in order to have a standardized inclusion criterium.

The results of the mutational analysis will be available within 2 weeks, after which the patient can be included in the study.

The presence of mutations will be screened for by DGGE method on exons 18 through 21 and sequencing analysis of exon 19 and 21. The exons examined will be those in which previously mutations have been found (9, 10). This analysis might be extended when additional mutation data become available.

The result needed for inclusion in the study will be based on the highly reliable DGGE methodology and will be delivered within 2 weeks of arrival of the sample at the laboratory.

All mutations will be confirmed by automatic sequencing. In case of (unlikely) non-confirmation of the DGGE result by the subsequent mutation analysis, the patients can continue with the treatment if the clinician judges this to be in the interest of the patient. However such patients will not be included in the final efficacy analysis and will therefore be replaced.

## Additional biomarkers

Determination of additional biomarkers will be assessed if other scientific evidence indicating their potential relevance with regard to the EGFR inhibition becomes available.

Such additional biomarkers including the amount of EGFR/pEGFR, HER2/neu receptor, Erk/pErk will be studied in the laboratory of experimental pathology (EXPA), VUB and other participating labs with specific expertise.

# Documentation and use of study data

## Documentation of study data

A case report form (CRF) will be provided for every patient as a computerized data base record (PDF).

A hard copy of the CRF will be generated by printing the completed pages of the computerized CRF.

The participating investigator, or designated representative, should complete the CRF as soon as possible after the information is collected. The completed CRF must be reviewed and signed by the investigator named in the clinical study protocol or by a designated sub-investigator.

## Intellectual property, authors rights and publications

This is an investigator initiated trial. Its scientific data are the property of all the investigators and of its study coordinators more specifically. No data can be released at any occasion without the prior approval of each of its study coordinators. The following rules guide the authorship of its manuscripts:

All manuscripts and abstracts dealing with the results of the phase II study will mention the names of all the study coordinators and those investigators who contributed at least 10% of evaluable patients. Those institutions contributing 20% or more of the evaluable patients can access to a second authorship. The sequence of the authorship will be according to the respective accrual of the participating institution, the first and corresponding author being the principal investigator and the last author one of the study coordinators.

If separate manuscripts on technical aspects of the mutational analysis or PET scan results would be submitted, it will contain the names of the study coordinators and of all the investigators involved in those technical aspects, according to the respective contribution to the success of the study. Here, the first author will be the investigator who has actually written the manuscript and the last and corresponding author the principal investigator of the overall study.

The principal investigator has final responsibility in applying these provisions and in writing up the main study result.

# Ethical and legal aspects

## Good clinical practice (GCP)

This study is to be conducted according to the globally accepted standards of good clinical practice (as defined in the ICH E6 Guideline for Good Clinical Practice, 1 May 1996), in agreement with the latest revision of the declaration of Helsinki and in keeping with local regulations.

## Patient study-files

Individual patient-study files (clinical report files, CRF) will be established in parallel to the hospital patient file. The CRF-file will exist as a computer-file (in MS Access) and a hard copy. The hard copy will be retained at the data management office at the treating medical center. A hard copy will be retained for at least 15 years after the completion of the study.

## Patient Informed Consent

Prior to the screening evaluation, the patient will be informed of the nature of the study drugs and procedures and will be given pertinent information as to the intended purpose, possible benefits, and possible adverse experiences. The procedures and possible hazards to which the patient will be exposed will be explained. Two approved informed consent statements, one for the mutation analysis and one for the treatment phase will then be read and signed by the patient, and, when required, a witness, and the investigator. The patient will be provided with a copy of the signed informed consent statements. The patient may withdraw from the study at anytime without prejudicing future medical treatment. Verification of the signed informed consent statements will be noted on the patient's study case report form.

## Ethical Committee/Institutional Review Board

The final approved protocol and the informed consent statements will be reviewed by a properly constituted Ethics Committee/IRB. The Ethics Committee's/Board's decision concerning the conduct of the study will be made in writing to the investigator.

The investigator will make required progress reports to the Ethics committee/IRB, as well as report any treatment related deaths.

The investigator will inform the Ethics Committee/IRB by of the termination of the study.

## Ethical Considerations

Eligible patients will be asked if they wish to participate in the study. The outline of the study will be explained by one of the staff physicians. In case the patient refuses to participate this will be respected and "standard" treatment will be offered.

## Insurance

The insurance has been taken by the sponsor (AZ-VUB) with regard to this study protocol, in order to cover all participating investigators, co-investigators and staff involved in this academic, non-commercial trial.

## Modification of the protocol

Any modifications to the protocol which may impact on the conduct of the study, potential benefit of the study, or may affect patient safety, including changes of study objectives, study design, patient population, sample sizes, study procedures, or significant administrative aspects will be made by a formal amendment to the protocol. The Ethics Committee/IRB will approve such amendment prior to implementation.

# Study monitoring

The central datamagement of the study will collect the date from the clinical report files at regular intervals after contacting the collaborating investigators. The availability of a computerized CRF will facilitate the data transfer by email or post.

In case of on site monitoring of source documents, the participating investigators must ensure direct access to the on-site study documentation and medical records.

# Appendices

1. Serious adverse event (SAE) report fill out form.
2. Patient’s information sheets and informed consent.
3. WHO scale for performance status and correspondence with Karnofsky index
4. Common Terminology Criteria for Adverse Events v3.0 (CTCAE) Publish Date: June 10, 2003. Will be provided as a PDF file on CD-ROM.
5. CT and FDG scan specifications.

Concomitant medication list excluding participation (CYP 3A4 inhibitors-inducers)

# 19 Appendix 1 SAE reporting form

# 19 Appendix 2 Informed Consent

Undersigned, …………………, declares to have received oral information on the study entitled:

“**PROSPECTIVE EVALUATION OF SMALL MOLECULE EGFR-1 TYROSINE KINASE INHIBITION AS A FIRST-LINE TREATMENT IN A PHASE II STUDY IN PATIENTS WITH ADVANCED NON-SMALL CELL LUNG CANCER (NSCLC) HARBOURING A MUTANT EGFR GENE ”**

By my physician, dr ………………….. .

I understand that for the stage of lung cancer with which I have been diagnosed that there is a standard of care consisting of systemic chemotherapy. This treatment can significantly extend my survival and quality of life when compared to having only treatments to control the symptoms of my disease but without chemotherapy.

If I agree to participate in the aforementioned study, I will receive an oral drug, called a tyrosine kinase inhibitor (erlotinib).

In order to verify my eligibility for this study, a agree that a genetic analysis is performed on my tumour biopsy in order to determine whether a specific gene, the epidermal growth factor receptor gene 1 or EGFR carries mutations that result in a protein with which the drug erlotinib interacts. If my tumour cells do not have these mutations, then I will not enter the study and receive the standard treatment for my disease.

This treatment is being proposed to me as an alternate to standard chemotherapy as an initial therapy. However if the study drug would fail to control my disease, I will leave the study and be offered standard treatment consisting of chemotherapy.

The assessment of efficacy or failure of the treatment will be based on my disease symptoms, clinical evaluation by my physician and the exams which are planned in the course of the study.

The study is being proposed to me if my cancer cells contain a mutant cancer gene that generates a mutant protein on the surface of the cancer cells, the Epidermal Growth Factor Receptor or EGFR.

In prior studies it has been shown that the cancers carrying such a mutation might be highly sensitive to erlotinib. However the proportion of patients responding to this type of drug is currently undefined.

Erlotinib is a medication that is taken orally and daily. The drug is generally well tolerated.

Common side effects of the drug that have been observed in earlier studies and in routine compassionate use programs are skin rash, acne and diarrhoea.

The current study aims at determining the percentage of patients benefiting from this novel treatment.

These drugs are generally better tolerated than chemotherapy, which is the reason why they are being explored in a first-line setting.

There are currently no data that suggest that having these new drugs first will influence my chances to benefit from subsequent standard chemotherapy if needed.

The different mechanism of action of these drugs and chemotherapy suggests that cross-resistance is unlikely. Moreover, these drugs have been given simultaneously with chemotherapy in large randomized studies without diminishing the effect of chemotherapy.

If I agree to participate in the study, I also agree that there will be a mutation analysis of the Epidermal Growth Factor Receptor (EGFR) and subsequent other studies on the cancer tissue that has been taken from me (biopsy) to make the diagnosis of my disease.

These additional studies will be performed to elucidate further the mechanisms why some patients do or do not respond to these treatments.

If I agree to participate in the study, I will have regular assessments of the effect of the treatment on the disease with CAT scans, PET scans and other exams deemed useful by my physician to evaluate the efficacy of the treatment. My physician has explained the nature of these exams to me. The exams will be performed before study initiation and thereafter 4 times with 6-week intervals. After 24 weeks on treatment, the exams will only be performed every 12 weeks, unless judged necessary earlier. I will in principle remain in the study as long as the evaluations and my clinical condition indicate that I benefit from the treatment.

I, the patient, can for any reason and at any time decide to leave the study and to continue with standard treatment or any other available option.

This study is being run and performed by university hospitals and large regional hospitals, independent from pharmaceutical companies. All the data that are collected during the study will be recorded in a file that will remain confidential and only accessible to my physician and his collaborators involved in my care and whom are all bound by medical secrecy.

My identity will never be disclosed to any outside entity or person.

My physician also will decide for me to leave the study, whenever he believes this to be in my interest.

Non-participation in this study will have no impact on the standard care I will receive as an alternative.

My participation in the study is covered by a specific medical insurance contracted by the hospital at which I am being treated.

The treatments and exams performed in addition to what normally would be required for my treatment are free, but I will receive no specific remuneration for participation in the study.

Dated Signature of the patient

…………………………..

Dated signature of physician

Copy of this signed informed consent for the patient.

**Schriftelijke toestemming en informatie.**

Ondergetekende, …………………………………………………… verklaart mondelinge informatie te hebben gekregen over de studie getiteld:

“**PROSPECTIVE EVALUATION OF SMALL MOLECULE EGFR-1 TYROSINE KINASE INHIBITION AS A FIRST-LINE TREATMENT IN A PHASE II STUDY IN PATIENTS WITH ADVANCED NON-SMALL CELL LUNG CANCER (NSCLC) HARBOURING A MUTANT EGFR GENE ”**

De uitleg over de studie werd mij gegeven door mijn arts,

dokter ………………………………………………………………………………….

Ik begrijp dat, voor het stadium van longkanker dat bij mij werd vastgesteld, normaal een behandeling met chemotherapie wordt voorgesteld. Dergelijke behandeling kan mijn overleving en levenskwaliteit bevorderen in vergelijking met wanneer ik enkel behandelingen zou krijgen die de symptomen van mijn ziekte zouden behandelen zonder chemotherapie.

In het kader van deze studie wordt mij echter voorgesteld eerst een nieuwe behandeling te krijgen waarvan verwacht wordt dat ze in mijn geval een belangrijk effect op de ziekte kan hebben. Het is op dit ogenblik echter nog niet precies geweten in welk percentage van patiënten een gunstig effect kan verwacht worden en hoelang zulk effect dan aanhoudt. Uit de al beschikbare informatie is gebleken dat de antitumor effecten van deze nieuwe behandeling in patiënten met een gelijkaardige ziekte als de mijne tot belangrijke effecten heeft geleid met soms langdurige ziekte remissies.

Evenmin als voor chemotherapie echter kan voorspeld worden of dit ook bij mij het geval zal zijn. Indien ik deelneem aan deze studie zal ik van nabij gevolgd worden met een aantal onderzoeken die regelmatig worden uitgevoerd zoals een CT-scan en een PET-scan. Indien blijkt dat deze behandeling niet het beoogde effect bereikt of op enige andere wijze best niet wordt verder gezet, zal door mijn arts de voor mijn toestand normale behandeling worden ingesteld.

Ik ben akkoord om deel te nemen aan de vermelde studie en ik zal bijgevolg een medicatie krijgen die langs de mond kan worden ingenomen en die erlotinib genoemd wordt. Dit geneesmiddel is een tyrosine kinase inhibitor en blokkeert de werking van een kankereiwit aangemaakt door een kankergen zonder hetwelk de kankercellen in vele gevallen zoals het mijne niet kunnen overleven. Het is door het blokkeren van dit kankereiwit dat een gunstig effect op mijn ziekte kan bekomen worden.

Om te kunnen nagaan of ik kan deelnemen aan deze studie ben ik akkoord om een staal te laten nemen van mijn tumor om een genetische analyse te kunnen uitvoeren. Deze genetische analyse moet toelaten om te bepalen of een specifiek gen, de epidermale groeifactor receptor gen 1 of EGFR een mutatie draagt die leidt tot de aanmaak van een eiwit waarop het geneesmiddel erlotinib inwerkt. In een vroegere studie is aangetoond dat kankers die zo een mutatie dragen erg gevoelig kunnen zijn aan erlotinib.

Als mijn kankercellen geen dergelijke mutatie hebben die het eiwit activeert zal ik niet aan de studie deelnemen en bijgevolg de normale behandeling voor mijn ziekte krijgen.

De behandeling wordt mij voorgesteld als een alternatief voor de normale chemotherapie als eerstekeuze behandeling. Als het geneesmiddel echter zou falen en mijn ziekte verergert zal ik de studie verlaten en ook de normale behandeling, bestaande uit chemotherapie, aangeboden krijgen.

De werkzaamheid of het falen van de behandeling zal worden beoordeeld op basis van mijn klachten, de klinische beoordeling door mijn arts en de onderzoeken die voorzien zijn in de loop van de studie op bepaalde tijdsstippen. Als ik instem om deel te nemen aan de studie zal ik regelmatig geplande onderzoeken hebben om het effect van de behandeling op mijn ziekte na te gaan met behulp van CAT-scans, PET-scans en eventueel andere onderzoeken die nuttig worden gevonden om het nut van de behandeling bij mij na te gaan.

Deze onderzoeken worden gedurende de eerste 6 maand om de 6 weken uitgevoerd en nadien om de 3 maand.

Erlotinib wordt dagelijks langs de mond ingenomen. Het geneesmiddel wordt goed verdragen. De meest belangrijke bijwerkingen die werden vastgesteld in vroegere studies en ook al in “compassionate use” programma’s zijn: huiduitslag, acné en diarree. In de huidige studie wil men bepalen welk percentage van de patiënten met mijn ziekteprofiel voordeel kunnen halen uit deze behandeling. Dit type geneesmiddel is over het algemeen beter verdragen dan chemotherapie hetgeen ook de reden is waarom de toepassing als eerstekeuze wordt onderzocht. Er zijn op dit ogenblik geen gegevens die zouden suggereren dat het krijgen van deze nieuwe behandeling voor chemotherapie mijn kans op een gunstig effect van daaropvolgende chemotherapie zal verminderen. De verschillende manieren van werken van deze geneesmiddelen in vergelijking met chemotherapie maakt een kruisweerstand onwaarschijnlijk. Bovendien zijn deze geneesmiddelen in grote fase III studies al onderzocht en daarin kon vastgesteld worden dat het effect van de chemotherapie niet verminderd werd. Ook niet wanneer deze medicatie samen met de chemotherapie worden toegediend.

Indien ik toestem om deel te nemen aan de studie sta ik ook toe dat er een mutatie analyse gebeurt op het EGFR gen ofwel op de bioptie die al genomen werd voor het stellen van de diagnose van mijn ziekte ofwel op een nieuwe bioptie indien het al afgenomen staal onvoldoende zou blijken. Ik stem er ook mee in dat verder wetenschappelijk laboratoriumonderzoek zou gebeuren op de bioptiën die bij mij werden afgenomen. Deze bijkomende onderzoeken zullen gebeuren om verder uit te maken waarom sommige patiënten wel en andere niet antwoorden op deze nieuwe behandelingen. Indien de ziekte zou hervallen onder deze behandeling dan zal mijn toestemming ook gevraagd worden om eventueel een nieuwe bioptie voor onderzoek van mijn ziekte te nemen. Dit onderzoek moet de mechanismen van weerstand aan de behandeling aan het licht brengen. Ik ben vrij om hier al dan niet op in te gaan.

Ik blijf in principe in de studie zolang de onderzoeken en mijn toestand aangeven dat ik voordeel haal uit de behandeling. Ik, de patiënt, kan voor om het even welke reden en om het even welk ogenblik, beslissen om de studie te verlaten en verder te gaan met de normale standaard behandeling of andere behandelingen of opties die nuttig kunnen zijn voor mij. Deze studie wordt geleid en uitgevoerd door universitaire ziekenhuizen en grote regionale ziekenhuizen, onafhankelijk van de farmaceutische industrie. Al de gegevens die verzameld worden tijdens deze studie worden opgenomen in een vertrouwelijk dossier dat enkel toegankelijk is voor mijn arts en zijn medewerkers die ook betrokken zijn in de zorg alsook de onderzoekers en die alle gehouden zijn aan het medische geheim. Mijn identiteit zal nooit worden kenbaar gemaakt aan een instelling of personen buiten de instelling waarin ik verzorgd word. Ook mijn arts zal beslissen dat ik de studie verlaat wanneer hij denkt dat dit in mijn voordeel is. Als ik niet deelneem aan deze studie zal dat geen enkele invloed hebben op de normale zorg die ik als alternatief zal krijgen.

Mijn deelname aan de studie is gedekt door een specifieke medische verzekering. De bijzondere onderzoeken en behandelingen die ik krijg in het kader van de studie en die meer zijn dan wat normaal voor mijn toestand noodzakelijk is worden gratis verricht. Ik zal echter geen vergoeding krijgen voor deelname in de studie.

Handtekening patiënt Datum

Handtekening arts Datum

Kopij van deze geschreven toelating wordt aan de patiënt overhandigd.

**Consentement informé et écrit**

Le la soussigné, Mr, Mme, …………………………………

déclare avoir reçu les informations orales sur l'étude nommée :

PROSPECTIVE EVALUATION OF SMALL MOLECULE EGFR-1 TYROSINE KINASE INHIBITION AS A FIRST-LINE TREATMENT IN PATIENTS WITH ADVANCED NON-SMALL CELL LUNG CANCER (NSCLC) HARBOURING A MUTANT EGFR GENE

L'information m'a été fournie par mon médecin, le docteur………………………

Je comprends que pour le stade de cancer des poumons qu'on a diagnostiqué chez moi, les traitements normaux contiennent de la chimiothérapie. Ce traitement peut prolonger ma vie et augmenter ma qualité de vie en comparaison avec un traitement purement symptomatique sans chimiothérapie.

Dans le cadre de l'étude qui m'a été proposée je serais traité en premier lieu par un nouveau médicament dont on attend un effet majeur possible sur ma maladie. Actuellement il n'est pas encore précisé dans quel pourcentage de patients un effet bénéfique peut être obtenu et quel sera la durée d'un effet bénéfique. Des informations disponibles il paraît que l’effet anti-tumoral de ce nouveau traitement chez des patients avec une maladie similaire que la mienne a induit des remissions, parfois de longue durée. Comme pour la chimiothérapie il n'est pas possible de prédire si ce sera le cas chez moi. Si je participe à l'étude je serais suivi de près avec un nombre d'examens qui seront répétés régulièrement, incluant un CT-scan et un PET-scan. S'il apparaît que le traitement n'a pas l'effet désiré ou qu'il y a une autre raison pour lequel le traitement ne peut être continué, mon médecin va instituer ou proposer un traitement établi pour ma condition. Je suis d'accord pour participer à l'étude et je recevrais donc la médication qui est pris par voie orale et qui s'appelle erlotinib. Ce médicament est un inhibiteur de la tyrosine kinase et bloque l'action d'une protéine produite par un oncogène et sans laquelle les cellules cancéreuses ne peuvent pas survivre dans beaucoup de cas. Le blocage de cette oncoproteïne pourrait donc être responsable pour un effet favorable sur ma maladie. Avant de pouvoir participer à l'étude il faut vérifier si je suis éligible. Pour cela je suis d'accord pour qu'un échantillon de ma tumeur soit utilisé pour une analyse génétique.

A cette fin la biopsie qui a aussi été utilisée pour faire mon diagnostique sera utilisée. L'analyse génétique doit permettre de savoir si un gène spécifique, le epidermal growth factor receptor gene-1 ou EGFR, contient une mutation, produisant ainsi la proteine sur laquelle le médicament Erlotinib agit. Dans des études précédentes il est démontré que des cancers qui portent une mutation dans ce gène peuvent être très sensibles à l'Erlotinib. Si une mutation n'est pas retrouvée dans ce gène dans les cellules cancéreuses je ne pourrais pas participer à l'étude et recevrais le traitement prévu normalement pour ma maladie.

Si, pendant l’étude, le médicament ne marche plus et que ma maladie s'aggrave, je quitterais l'étude et on me proposera le traitement normal. Le succès du traitement sera jugé à base de mes plaintes, les observations cliniques de mon médecin et les examens qui sont prévus dans le cours de l'étude à des moments prédéterminés.

Si je participe à l'étude ces examens seront effectués régulièrement : CT-scan, PET-scan, supplémentés éventuellement par d'autres examens qui pourraient être jugés utiles pour évaluer l'effet du traitement. Les examens sont effectués tous les 6 semaines pendant les premiers 6 mois et après tous les 3 mois. L'Erlotinib est pris oralement, une fois par jour. Le médicament est bien supporté. Les effets secondaires les plus importants qui ont été démontrés dans des études antérieures et aussi dans des programmes d’utilisation compassionnelle sont: éruption cutanée, acné et diarrhée. Le médicament est en général mieux toléré que la chimiothérapie, ce qui est une des raisons pour lesquelles l'application comme traitement de première ligne est investigué. Le bût de l'étude est déterminer le pourcentage de patients bénéficière de ce traitement. En ce moment il n'y a pas de donnés qui suggèrent que les patient qui ont reçu ce nouveau traitement avant la chimiothérapie diminuerait l'effet d'une chimiothérapie secondaire. Le mode d'action de ce nouveau médicament en comparaison avec le mode d'action de la chimiothérapie rend peu probable un mécanisme de résistance commun. En plus ce médicament a déjà été testé dans des grandes études comparatives (phase III) et dans lesquelles ont a constaté que l'effet de la chimiothérapie n'est pas diminué en association avec l'Erlotinib. Même pas si l'Erlotinib est donné en même temps que la chimiothérapie. Si je consens de participer dans l'étude je suis d'accord pour la recherche de mutation dans le gène EGFR sur une biopsie qui a déjà été prise pour des buts diagnostiques ou bien à partir d'une nouvelle biopsie si le premier échantillon est insuffisant. Je suis aussi d'accord que d’autres examens scientifiques ultérieurs soient continués sur mes échantillons. Un des buts de ces examens complémentaires serait de déterminer pourquoi certains patients répondent et d'autres pas au nouveau traitement. Si ma maladie récidiverait sous traitement avec l'Erlotinib on me demandera une autorisation de prendre une nouvelle biopsie pour essayer de détecter la cause de la résistance au traitement. Je suis tout à fait libre de consentir ou pas à cette nouvelle biopsie. Je reste en principe dans l'étude aussi longtemps que les examens et mon état indiquent que je bénéficie de ce traitement. Moi, comme patient, je peux pour n'importe quelle raison et à n'importe quel moment décider de quitter l'étude et de continuer avec un traitement normal ou d'autres traitements ou options qui pourraient être utile pour moi. Cette étude est menée et exécutée par des médecins-investigateurs hôpitaux universitaires et des grands hôpitaux régionaux, indépendant de l'industrie pharmaceutique. Toutes les données qui sont collectées pendant l'étude sont rassemblés dans un dossier confidentiel qui est seulement accessible au médecin et ses collaborateurs et qui sont impliqués dans mes soins ainsi que les investigateurs qui sont tous tenus au secret médical. Mon identité ne sera jamais divulgué à une institution ou à des personnes en dehors de l'institution où je suis soigné. Mon médecin aussi décidera que je quitte l’étude dès qu'il pense que ce serait dans mon avantage. Si je décide de ne pas participer à cette étude cette décision n’aura aucune influence sur les soins normaux que je recevrais comme alternative. Ma participation dans l'étude est couverte par une assurance médicale spécifique. Le traitement que je recevrais, dans le cadre de l'étude ainsi que les examens qui sont faits spécifiquement pour l’étude seront gratuit. Je ne reçois aucune récompense pour participer à l'étude en dehors du médicament.

Date et signature du patient

Date et signature du médecin

Une copie de consentement écrit est transmis au patient.

# 19 Appendix 3 WHO - KPS

| WHO-SCALE [Grade] | | KARNOFSKY-SCALE [%] | |
| --- | --- | --- | --- |
| 0 | Able to carry out all normal activity without restriction. | 100 | Normal, no complaints |
| 1 | Restricted in physically strenuous  activity by ambulatory and able to carry out light work. | 90 | Able to carry on normal activities; minor signs or symptoms of disease |
|  |  | 80 | Normal activity with effort. |
| 2 | Ambulatory and capable of all  self-care but unable to carry out  any work; up and about more than 50 % of waking hours. | 70 | Cares for self. Able to carry on normal activity or to do active work. |
|  |  | 60 | Ambulatory. Requires some assistance in activities of daily living and self-care. |
| 3 | Capable of only limited self-care;  confined to bed or chair more than 50 % of waking hours. | 50 | Requires considerable assistance of frequent medical care. |
|  |  | 40 | Disabled; requires special care and assistance. |
| 4 | Completely disabled; cannot carry on any self-care: totally confined to bed or chair. | 30 | Severely disabled; hospitalization indicated though death not imminent. |
|  |  | 20 | Very sick; hospitalization and active supportive treatment. |
|  |  | 10 | Moribund. |
|  |  | 0 | Dead. |

# 19 Appendix 4 CTC AE 3.0

# 19 Appendix 5 Imaging Specifications

All CT-scan performed within this study will be spiral CT-scans.

### PET-scan for evaluation of metabolic response

Patient preparation

- 6 hours fasting (postpone patient if not fasted)
- valium p.o. prophylaxis if needed
- diabetes patients under peroral treatment: overnight fasting; no peroral antidiabetics in the morning; plan patient early in morning; if glycemia > 140 and < 200 mg% 🡺 follow scheme here under.
- in insuline-dependent diabetes patients: plan patient early in the morning after overnight fasting; no insuline in the morning.

-if fasting glycemia < 140 mg% 🡺 FDG injection;

-if fasting glycemia > 200 mg/dl: 🡺 exclude patient

-if > 140 mg/dl et < 200 mg/dl 🡺 administration of IV Insuline and monitor glycemia until under 140 mg%.

Image acquisition

- start imaging at 60 minutes (+/- 5 minutes) post FDG administration
- whole body post-injection transmission scan or, CT (in case of PET-CT camera) for attenuation correction
- note body weight / length / glycemia

Image analysis

- visual analysis: 3 points score: normal, equivocal, pathological
- response analysis (always in relation to the baseline, pre-treatment, PET-imaging): 4 points score according to the criteria defined by the EORTC PET study group for assessing response using FDG18
  - Progressive metabolic disease
    - Increase in SUV of more than 25%
    - Visual increase in lesion extent (>20% in longest dimension)
    - New metastatic lesions
  - Stable metabolic disease
    - Increase in SUV < 25%, decrease < 15%
    - No visible increase in the extent of FDG uptake
  - Partial metabolic response
    - Reduction in SUV between 15 and 25% after 1 cycle of treatment
    - Reduction in SUV > 25% after more than 1 cycle of treatment
  - Complete metabolic response
    - Complete resolution of FDG-uptake

# 20 References

1. Bunn PA, Jr.: Chemotherapy for advanced non-small-cell lung cancer: who, what, when, why? J Clin Oncol 20:23S-33S, 2002
2. Fukuoka M, Yano S, Giaccone G, et al: Multi-institutional randomized phase II trial of gefitinib for previously treated patients with advanced non-small-cell lung cancer. J Clin Oncol 21:2237-46, 2003
3. Smit EF, van Meerbeeck JP, Lianes P, et al: Three-arm randomized study of two cisplatin-based regimens and paclitaxel plus gemcitabine in advanced non-small-cell lung cancer: a phase III trial of the European Organization for Research and Treatment of Cancer Lung Cancer Group--EORTC 08975. J Clin Oncol 21:3909-17, 2003
4. Kris MG, Natale RB, Herbst RS, et al: Efficacy of gefitinib, an inhibitor of the epidermal growth factor receptor tyrosine kinase, in symptomatic patients with non-small cell lung cancer: a randomized trial. Jama 290:2149-58, 2003
5. F.A. Shepherd et al [J. Pereira, T.E. Ciuleau, E.H. Tan, V. Hirsh, S. Thougprasert, A. Bezjak, D. Tu, P. Santabarbara and L. Seymour] A randomized placebo-controlled trial of erlotinib in patients with advanced non-small-cell lung cancer (NSCLC) following failure of 1st line of 2nd line chemotherapy. A National Cancer Institute of Canada Clinical Trials Group (NCIC CTG) trial. Proceedings ASCO 2004, 7022, 18
6. N.R. Dickson et al Single agent gefitinib in poor performance status patients with previously untreated advanced non-small-cell lung cancer: A Minnie Pearl Cancer Research Network Phase II trial Proceedings ASCO 2004 7086 p 634
7. Herbst RS, Giaccone G, Schiller JH, et al: Gefitinib in combination with paclitaxel and carboplatin in advanced non-small-cell lung cancer: a phase III trial--INTACT 2. J Clin Oncol 22:785-94, 2004
8. Giaccone G, Herbst RS, Manegold C, et al: Gefitinib in combination with gemcitabine and cisplatin in advanced non-small-cell lung cancer: a phase III trial--INTACT 1. J Clin Oncol 22:777-84, 2004
9. Paez JG, Janne PA, Lee JC, et al: EGFR mutations in lung cancer: correlation with clinical response to gefitinib therapy. Science 304:1497-500, 2004
10. Lynch TJ, Bell DW, Sordella R, et al: Activating mutations in the epidermal growth factor receptor underlying responsiveness of non-small-cell lung cancer to gefitinib. N Engl J Med 350:2129-39, 2004

Pham D., Kris M. G., McDonough T., Riely G. J., Venkatraman E. S., Pao W., Wilson R. K., Miller V. A., Singh B., Rusch V. W.. Estimation of the likelihood of epidermal growth factor receptor (EGFR) mutations based on cigarette smoking history in patients with adenocarcinoma of the lung Proceedings ASCO 2005, 7069.

1. Khuri FR, Jain SR: Novel agents and incremental advances in the treatment of head and neck cancer. Semin Oncol 31:3-10, 2004
2. Cunningham D, Humblet Y, Siena S, et al: Cetuximab Monotherapy and Cetuximab plus Irinotecan in Irinotecan-Refractory Metastatic Colorectal Cancer. N Engl J Med 351:337-345, 2004
3. Starling N, Cunningham D: Monoclonal antibodies against vascular endothelial growth factor and epidermal growth factor receptor in advanced colorectal cancers: present and future directions. Curr Opin Oncol 16:385-90, 2004
4. Govindan R: Cetuximab in Advanced Non-Small Cell Lung Cancer. Clin Cancer Res 10:4241S-4244, 2004
5. Pao W, Miller V, Zakowski M, et al: EGF receptor gene mutations are common in lung cancers from "never smokers" and are associated with sensitivity of tumors to gefitinib and erlotinib. Proc Natl Acad Sci U S A 101:13306-11, 2004

Miller V. A., Zakowski M., Riely G. J., Pao W., Hussain S., Ladanyi M., Heelan R. T., Kris M. G. EGFR mutation, immunohistochemistry (IHC) and chromogenic in situ hybridization (CISH) as predictors of sensitivity to erlotinib and gefitinib in patients (pts) with NSCLC Proceedings ASCO 2005, 7031

Tsao M. S., Sakurada A., Lorimer I., Cutz J., Kamel-Reid S., Squire J., Ding K., Frank R., Seymour L., Shepherd F. Molecular analysis of the epidermal growth factor receptor (EGFR) gene and protein expression in patients treated with erlotinib in National Cancer Institute of Canada Clinical Trials Group (NCIC CTG) trial BR.21. Proceedings ASCO 2005, 7007

1. Perez-Soler R, Chachoua A, Hammond LA, Rowinsky EK, Huberman M, Karp D, Rigas J, Clark GM, Santabarbara P, Bonomi P. Determinants of tumor response and survival with erlotinib in patients with non--small-cell lung cancer. J Clin Oncol. 2004 Aug 15;22(16):3238-47.
2. Gatzemeier U., Pluzanska A., Szczesna A., Kaukel E., Roubec J., Brennscheidt U., De Rosa F., Mueller B., Von Pawel J., for the TALENT Study Investigators Results of a phase III trial of erlotinib (OSI-774) combined with cisplatin and gemcitabine (GC) chemotherapy in advanced non-small cell lung cancer (NSCLC). Journal of Clinical Oncology, 2004 ASCO Annual Meeting Proceedings (Post-Meeting Edition). Vol 22, No 14S (July 15 Supplement), 2004: 7010
3. Herbst R. S., Prager D., Hermann R., Miller V., Fehrenbacher L., Hoffman P., Johnson B., A. B.Sandler, Mass R., Johnson D. H.; TRIBUTE - A phase III trial of erlotinib HCl (OSI-774) combined with carboplatin and paclitaxel (CP) chemotherapy in advanced non-small cell lung cancer (NSCLC). Journal of Clinical Oncology, 2004 ASCO Annual Meeting Proceedings (Post-Meeting Edition). Vol 22, No 14S (July 15 Supplement), 2004: 7011
4. Shepherd FA, Rodrigues Pereira J, Ciuleanu T, Tan EH, Hirsh V, Thongprasert S,Campos D, Maoleekoonpiroj S, Smylie M, Martins R, van Kooten M, Dediu M, Findlay B, Tu D, Johnston D, Bezjak A, Clark G, Santabarbara P, Seymour LN. Erlotinib in previously treated non-small-cell lung cancer. Engl J Med. 2005 Jul 14;353(2):123-32;
5. Therasse P, Arbuck SG, Eisenhauer EA, et al: New guidelines to evaluate the response to treatment in solid tumors. European Organization for Research and Treatment of Cancer, National Cancer Institute of the United States, National Cancer Institute of Canada. J Natl Cancer Inst 92:205-16, 2000
