# Supplementary material for: Prospective Evaluation of First-Line Erlotinib in Advanced Non-Small Cell Lung Cancer (NSCLC) Carrying an Activating EGFR Mutation: A Multicenter Academic Phase II Study in Caucasian Patients (FIELT)
Source: PLoS One. 2016 Mar 31;11(3):e0147599. doi: 10.1371/journal.pone.0147599 (PMC4816447; doi:10.1371/journal.pone.0147599)
Supplement: S2 Text — (PDF) [file pone.0147599.s002.pdf]

ACADEMISCH ZIEKENHUIS – VRIJE UNIVERSITEIT BRUSSEL

COMMISSIE MEDISCHE ETHIEK (O.G. 016)
Reflectiegroep Biomedische Ethiek
Laarbeeklaan 101 B - 1090 BRUSSELS BELGIUM
mdrsnm@az.vub.ac.be
Tel. 00 32 2 477 55 84
Fax 00 32 2 477 55 94

Uw Kenmerk :
Ons Kenmerk : 2005/[illegible]
(Gelieve in uw verdere correspondentiesteeds onze referentie te hernemen aub)

Prof. dr. J. De Grève and dr. D. Schallier
Oncology
AZ-VUB

Jette, 12 January 2006

LEADING ETHICS COMMITTEE – SINGLE OPINION FORM

Concerning: trial :

FIELT STUDY : First line Inhibitor of EGFR in Lung cancer Treatment.
Multicenter study – prospective evaluation of small molecule EGFR-1 tyrosine kinase inhibition as a first-line treatment in patients with advanced non-small cell lung cancer (NSCLC) harbouring a mutant EGFR gene.
Protocol VUB05-002, version 2.0 dd. 26.10.2005 – AZ-VUB
EUDRACTN° 2005-003519-57

The Medical Ethics Committee has examined the following documents concerning the above mentioned project :

- " Adviesaanvraagformulier aan de Commissie Medische Ethiek voor een project voor experiment bij de mens"
- National Application for Clinical Trial
- List of non-leading EC' s and list of participating investigators
- Protocol VUB 05-002 version 2.0 dd. 26/10/2005 and Protocol Synopsis
- Patient Information Sheets and Informed Consent Forms
  - in Dutch, French and English, **all versions 3.0 dated 27/12/2005, adapted according to our Ethics Committee remarks of 01/12/2005**
  - in German, version 1.0 dd dated 27/12/2005

  and Patient Prescreening Information Sheets and Informed Consent Forms in Dutch, French and English, **all versions 3.0 dated 27/12/2005, adapted according to our Ethics Committee remarks of 01/12/2005**
- Investigator' s brochure of Tarceva ™ dated 4 April 2005
- Financial Agreement signed by prof. dr. J. De Grève
- C.V.' s of the investigators
- Certificate of insurance by Ethias dated 16/09/2005

from the ethical, legal and medical science points of view and in accordance with the Belgian Law for experiments on humans of 7th May 2004, Chapter VIII – Article 11 – § 4 : items 1 till 11,

and has consulted the non-leading Ethics Committees (see enclosed list), in accordance with the Belgian Law for experiments on humans of 7th May 2004, for the items mentioned in Chapter VIII – Article 11 – § 4 : items 4, 6 and 7.

The AZ-VUB Ethics Committee, during its meeting on 12 January 2006,decides that :

THERE ARE NO OBJECTIONS TO THE STUDY IN ITS PRESENT FORM

THIS ADVICE TAKES INTO ACCOUNT THE REMARKS OF THE LOCAL COMMITEES OF:

- UZ Gent ✓
- UZ Leuven ✓
- CH Notre-Dame Charleroi ✓
- UC Louvain ✓
- ULB Erasme ✓
- AZ Middelheim en ZNA ✓
- CHR de la Citadelle ✓
- Institut Bordet ✓
- CHC Liège
- KLINA ✓
- Clinique St-Pierre Ottignies ✓
- Cliniques du Sud Luxembourg ✓

FOLLOWING LOCAL ETHICS COMMITTEES GAVE A FAVOURABLE. ADVICE:

- St.Niklaushospital Eupen ✓

THIS SINGLE OPINION DOES NOT TAKE INTO ACCOUNT THE ADVICE OF THE LOCAL ETHICS COMMITTEE OF:

- UZ Antwerpen ✓

WHO DID NOT GIVE HER REMARKS WITHIN THE LEGAL DEADLINE.

This approval is valid for the duration of the trial. The Committee wishes to obtain an annual trial status. Results of the trial should be transmitted to the Committee at the end of the trail (publication or summary if there is to be no publication). The Committee reminds the investigator of his personal responsibility for this project. The Committee does not take any responsibility for its favourable advice of the project. The Ethics Committee is organised and operates according to GCP and its applicable laws and regulations. The list of members who participated to the vote and who were present at the meeting is annexed.

P. Devroey,chairman

Cc : Local EC' s : see enclosed list
: Directoraat Generaal Geneesmiddelen, Departement Onderzoek en Ontwikkeling, Bisschoffsheimlaan 33 - 1000 Brussel

LIST NON-LEADING EC'S REF 2005/122.

AZ Klina
Augustijnlei 100
2930 Brasschaat
03/650.50.00

AZ Middelheim
Lindendreef 1
2020 Antwerpen
03/281.37.48

CH Notre-Dame et Reine Fabiola
Av. du Centenaire 73
6061 Montignies-sur-Sambre
071/27.85.01

~~CHL Luxembourg~~
~~Rue Barblé 4~~
~~1210 Luxembourg~~
~~0035 2 458 762~~
doet niet meer mee.

CHR de la Citadelle
Bld. du 12ieme de Ligne 1
4000 Liège
04/223.88.73

CHR St. Vincent
Rue Fr. Lefebvre 207
4000 Rocourt
04/239.41.12

Clinique St. Pierre
Av. Reine Fabiola 9
1340 Ottignies
010/41.49.53

Cliniques du Sud-Luxembourg
Rue des Déportées 137
[illegible]700 Arlon
[illegible]63/23.17..61

Hopital Erasme
Route de Lennik 808
1070 Bruxelles
02/555.46.20

Institut Jules Bordet
Rue Heger Bordet 1
1000 Bruxelles
02/541.37.95

St. Nikolaus Hospital
Hufengasse 4-8
4700 Eupen
087/59.92.98

UCL St. Luc
Av. Hippocrate 55-14
1200 Bruxelles
02/764.55.13

UZ Antwerpen
Wilrijkstraat 10
2650 Edegem
03/821.42.54

UZ Gasthuisberg
Herestraat 49
3000 Leuven
016.34.86.01

UZ Gent
De Pintelaan 185-2P4
9000 Gent
09/240.49.62

ACADEMISCH ZIEKENHUIS – VRIJE UNIVERSITEIT BRUSSEL

COMMISSIE MEDISCHE ETHIEK (O.G. 016)
Reflectiegroep Biomedische Ethiek

Laarbeeklaan 101 B – 1090 BRUSSELS
BELGIUM
mdrsnm@az.vub.ac.be

Tel. 00 32 2 477 55 84
Fax 00 32 2 477 55 94

12/01/2006

MEMBERS OF THE MEDICAL ETHICS COMMITTEE AZ-VUB (since 07/04/2005)

Name	Function	Gender	participated in vote	present at meeting of 12th January 2006
Prof. Dr. P. Devroey	**CHAIRMAN** Fertility AZ-VUB	M	●	●
Dr. J. Marchand	**VICE- CHAIRMAN** Pediatrics AZ-VUB	M	●	●
Dr. C. Baeken	Psychiatrics AZ-VUB	M	0	0
Prof. Dr. A. Bossuyt	Nuclear Medicine AZ-VUB	M	●	●
Prof. Dr. H. De Boeck	Pediatric Orthopedics AZ-VUB	M	●	●
Prof. Dr. J. De Grève	Medical Oncology and Hematology AZ-VUB	M	0	●
Prof. Dr. E. De Groot	General practioner and Lawyer – Tuyaertstraat 30, Boom	M	●	0
Mrs. M. De Win	Senior nurse and staff manager Nursing Education AZ-VUB	F	●	●
Mr. J. Foubert	Lecturer Erasmushogeschool Brussel – Laarbeeklaan 121, Jette	M	●	0
Prof. Dr. P. Haentjens	Orthopedics AZ-VUB	M	●	●
Apr. C. Ligneel	Pharmacy AZ-VUB	F	●	●
Prof. Dr. D. Roseeuw	Dermatology AZ-VUB	F	●	●
Dr. S. Yoshimi	General practioner Kasteellaan 130, Brussels	M	●	0

If member of the Ethics Committee, the principal investigator did not participate to the vote
